# Climate change could drive marine food web collapse through altered trophic flows and cyanobacterial proliferation

**DOI:** 10.1371/journal.pbio.2003446

**Published:** 2018-01-09

**Authors:** Hadayet Ullah, Ivan Nagelkerken, Silvan U. Goldenberg, Damien A. Fordham

**Affiliations:** 1 Southern Seas Ecology Laboratories, School of Biological Sciences, University of Adelaide, Adelaide, Australia; 2 The Environment Institute, School of Biological Sciences, The University of Adelaide, Adelaide, Australia; National Centre for Scientific Research (CNRS), France

## Abstract

Global warming and ocean acidification are forecast to exert significant impacts on marine ecosystems worldwide. However, most of these projections are based on ecological proxies or experiments on single species or simplified food webs. How energy fluxes are likely to change in marine food webs in response to future climates remains unclear, hampering forecasts of ecosystem functioning. Using a sophisticated mesocosm experiment, we model energy flows through a species-rich multilevel food web, with live habitats, natural abiotic variability, and the potential for intra- and intergenerational adaptation. We show experimentally that the combined stress of acidification and warming reduced energy flows from the first trophic level (primary producers and detritus) to the second (herbivores), and from the second to the third trophic level (carnivores). Warming in isolation also reduced the energy flow from herbivores to carnivores, the efficiency of energy transfer from primary producers and detritus to herbivores and detritivores, and the living biomass of detritivores, herbivores, and carnivores. Whilst warming and acidification jointly boosted primary producer biomass through an expansion of cyanobacteria, this biomass was converted to detritus rather than to biomass at higher trophic levels—i.e., production was constrained to the base of the food web. In contrast, ocean acidification affected the food web positively by enhancing trophic flow from detritus and primary producers to herbivores, and by increasing the biomass of carnivores. Our results show how future climate change can potentially weaken marine food webs through reduced energy flow to higher trophic levels and a shift towards a more detritus-based system, leading to food web simplification and altered producer–consumer dynamics, both of which have important implications for the structuring of benthic communities.

## Author summary

Healthy marine ecosystems are crucial for people’s livelihoods and food production. Global climate stressors, such as warming and ocean acidification, can drastically impact the structure and function of marine food webs, diminishing the production of goods and services. Our ability to predict how future food webs will respond to a changing environment is limited by our understanding of species responses to climate change, which are often tested in isolation or in simplified experimental designs. More realistic predictions of the impacts of climate change on ecosystems requires consideration of entire species communities, including the species interactions that can buffer or exacerbate these impacts. We experimentally tested the effects of warming and acidification, both individually and in combination, on a benthic marine food web in a near-natural ecological setting. Energy flow from the first trophic level (primary producers and detritus) to the second (herbivores), and from the second to the third trophic level (carnivores) was quantified under these different regimes. We show that warming, either alone or in combination with acidification, can constrain productivity to the bottom of the food web by enhancing cyanobacterial biomass and reducing energy flow to higher trophic levels, thus lowering energy transfer efficiency between producers and consumers. In contrast, increased ocean acidification alone showed a positive effect on herbivores and carnivores. Our finding is important because it demonstrates that future warming could drive marine food web collapses to potentially simplified and less productive coastal systems.

## Introduction

Forecasting the effect of global change on ecosystem functioning is a major challenge in ecology [1], partly because future climate shifts are likely to reorganize complex food webs, generating novel communities composed of new combinations of species [2]. In marine ecosystems, multiple anthropogenic stressors are already eroding biodiversity by changing the composition of species [3] and affecting rates of biomass transfer through ecological networks, resulting in altered food web organisation and dynamics [4,5]. While overexploitation is largely responsible for altering the structure and functioning of many ecosystems [6], global warming is forecast to amplify these effects, having serious consequences for the health and sustainability of marine ecosystems [7]. Despite many studies showing a potential detrimental effect of climate change on biodiversity [2], we still lack a strong and coherent theoretical and empirical foundation for understanding how species communities are likely to respond to global change [8].

Marine ecosystem functioning is maintained by the flow of energy from primary producers at the base of food webs through intermediate consumers to top predators, as well as via cycling of materials such as nutrients within the ecosystem [7,9]. Disturbances such as habitat modification can decouple, alter, or concentrate energy flows towards a smaller number of species and erode resilience by removing alternative feeding pathways in the food web [10]. This can drive trophic food webs to shift states and potentially collapse [11]. In this context, climate change can independently affect, or synergistically amplify, the effect of other disturbances such as habitat degradation, species overexploitation, and species invasions [12]. This can reconfigure future food webs through major structural changes, like shifts in the number of trophic groups and links that connect species at the top of the food chain to basal species, which can result in altered flows of energy and shifts in the biomass of key functional groups, leading to biodiversity loss, with potentially serious implications for ecosystem functioning [13].

Global warming and ocean acidification are already affecting the physiology, behaviour, phenology, demography, abundance, and distribution of many marine species [14]. Elevated temperature (*T*) affects fish performance and growth through increasing metabolic rates and respiratory demands, leading to a reduced aerobic scope for important life-supporting activities such as feeding, somatic growth, maturation, and predator avoidance [15–17]. Ocean acidification raises the energetic costs involved with calcification and acid-base regulation [18,19] and can impair neural functioning [20]. Besides such direct effects, there is a suite of indirect effects that can impact species persistence and diversity under global change [21].

The survival of a species or a group of species in an ecosystem depends on how well they respond to dynamic changes in productivity, in terms of direction (i.e., positive or negative) and strength. However, the responses of species to global change are not individual-based; they are connected through biotic interactions within and across trophic levels [22,23]. Importantly, the energy flow (biomass fluxes) to higher trophic levels is determined by various biological interactions (e.g., predator–prey relationships, competition, facilitation, and mutualism) of species that are directly or indirectly linked to proximate trophic levels [7,24]. For example, climate change can weaken the energy transfer efficiency between primary producers and consumers by reducing feeding performance with potential impacts further up the food chain [25]. Although ocean warming and acidification can boost basal productivity [26,27], it does not necessarily result in an increase in secondary productivity [28]. The propagation of production through trophic levels could be modified by food chain length [29] or with an increase in the dominance of herbivory-resistant primary producers [30,31]. Cyanobacteria, in the form of toxin-producing deleterious phytoplankton, can potentially divert productivity into alternate food web pathways, which are unavailable to higher trophic levels. Cyanobacteria have been forecast to increase in dominance as a result of global warming [29]. Conversely, in line with metabolic theory [32], increased food demand in predators can intensify top-down control of their prey populations [16], influencing body size-driven metabolic differences in food web structure [33]. Thus, a dichotomy between enhanced bottom up control through an increase in herbivory-resistant primary producers and enhanced top-down control due to higher metabolism-driven energetic demand in predators can jeopardise intermediate trophic levels and weaken food web stability. Such a mismatch can alter trophic energy flow dynamics [25,34], with the consequences expected to be greater in food webs with three trophic levels or more [29].

Forecasting the effects of climate change at the ecosystem level requires holistic approaches that consider complex ecological communities with multiple functional groups or species of different trophic levels [35]. Large-scale mesocosm experiments are ideal for such approaches and have the potential to enhance our understanding of the ecological consequences of climate change on the sudden expansion of opportunistic species, species extinction risk, community structure, and ecosystem functions [36].

We did a community-level manipulation of a temperate marine food web, consisting of 17 functional groups (ranging from primary producers to herbivores to carnivores across 3 broad trophic levels). We maintained these groups in an indoor mesocosm experiment divided into 4 treatments: elevated CO_2_ (*OA*), elevated temperature (*T*), elevated CO_2_ and temperature (*OAT*), and ambient controls (present-day levels of *p*CO_2_ and temperature), each with 3 replicate mesocosms per treatment. We achieved an elevated *p*CO_2_ of approximately 900 ppm (pH = 7.89) and warming of +2.8°C, which represent the conditions predicted for the end of this century, following a business-as-usual emission scenario (RCP8.5) [37]. We used an ecosystem modelling tool (Ecopath) widely used to characterise quantitative food web structures and pathways of energy flows in aquatic ecosystems [38]. The Ecopath model is built on a system of linear equations and creates a static mass-balanced snapshot of the resources in a given ecosystem according to biomass estimates and food consumption relationships of functional groups that represent the organisms in a food web. The quantitative description of food web properties is essential to advance our understanding of ecosystem structure and functioning at a community level [39]. Using the Ecopath approach, we tested whether: (1) global warming and ocean acidification enhance energy fluxes through bottom-up effects that stimulate primary productivity, or (2) global warming and ocean acidification allow opportunistc groups to proliferate and divert productivity into alternative pathways, as well as (3) biomass of lower trophic levels and detritus will dominate the future structure of marine food webs due to reduced energy transfer efficiencies to higher trophic levels. We also test whether synergies between ocean acidification and increasing temperature are likely to amplify the effect of global warming on marine ecosystems. Thus, through a combination of experimental and modelling approaches, we provide new evidence for altered energy flow (biomass fluxes) and transfer efficiency through food webs due to global change stressors, which is crucial for understanding the potential effects of climate change on marine food web structure and functioning.

## Results

**Fig 1:**
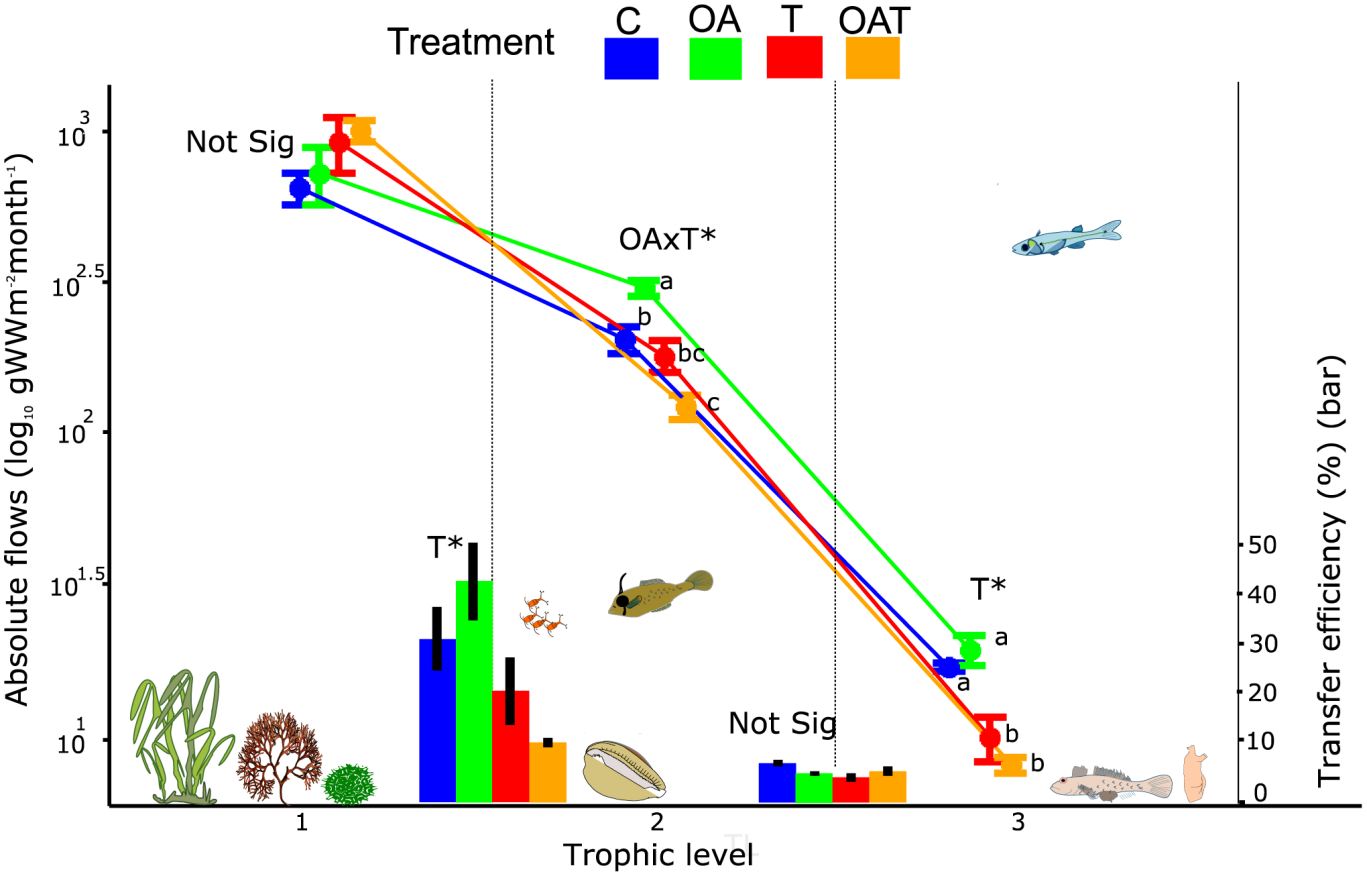
The effects of future climate on absolute flows and transfer efficiency between successive trophic levels of the mesocosm food web. Absolute flows, shown as line diagrams, refer to the total amount of energy that flows to higher trophic levels through consumption (log10 g Wet Weight/m^2^/month) aggregated by trophic level. The first trophic level shows flows originating from both the primary producers and detritus, which are transferred to the second and third trophic level through consumption by herbivores and carnivores, respectively. Transfer efficiency, presented as bar charts, refer to the ratio at which absolute flows are transferred from one trophic level to the next. Mean ± SE are based on *n* = 3 mesocosms. Significant effects (*p* < 0.05) within trophic levels are based on two-way ANOVAs (df = 1,8) and are indicated with asterisks. See S1 Table for statistical test outcomes. Means with different lower-case letters indicate significant differences among treatments based on posthoc tests corrected for false discovery rate and done separately for different trophic levels. The distribution of organisms within the mesocosms is reflected as their vertical position inside the graph (ranging from the bottom of the mesocosm to the surface of the water column). Species cliparts were obtained or modified from Openclipart (https://openclipart.org/). C, control; not sig, not significantly different; OA, elevated CO_2_; OAT, elevated CO_2_ and temperature; T, elevated temperature.

Neither warming nor acidification affected the energy flow originating from primary producers and detritus at trophic level 1 (Fig 1; S1 Table). The combined effect of warming and acidification (*OAT*) (*p* = 0.003; post hoc energy flow: *OAT* < control) reduced the energy flow from trophic level 1 to trophic level 2. In contrast, energy flow was higher in the *OA*-only treatment compared to the controls (*p* = 0.011; post hoc energy flow: high CO_2_ > control). Warming (*T* and *OAT*) also reduced the energy flows from trophic level 2 to trophic level 3 (ANOVA, F_1, 8_ = 43.06, *p* < 0.001).

The individual functional groups at trophic level 2 and trophic level 3 showed variable responses to warming and acidification. For example, functional groups such as meiobenthos, copepods, small epifaunal invertebrates, and filter feeders experienced significantly lower energy flow from trophic level 1 to trophic level 2 under warming (*T*), while macroinvertebrates experienced reduced flow under the combination of warming and acidification (*OAT*) (S1 Fig; S4 Table). Furthermore, warming significantly reduced the capacity of transferring energy flows of omnivorous, filter feeding, and benthic carnivorous fish, while benthic carnivorous and carnivorous fish experienced an increase in flow under acidification from trophic level 2 to trophic level 3 (S2 Fig, S5 Table).

The reduced energy flow from trophic levels 1 to 2 under the combined warming-acidification treatment (*OAT*) coincided with a negative effect of warming (*T* and *OAT*) on energy transfer efficiency between levels 1 and 2 (ANOVA, F_1,8_ = 11.22, *p* = 0.010) (Fig 1; S1 Table). In contrast, *OA* had no effect on energy transfer efficiency between these levels. Energy transfer efficiency between trophic levels 2 to 3 was not affected by either warming or acidification.

**Fig 2:**
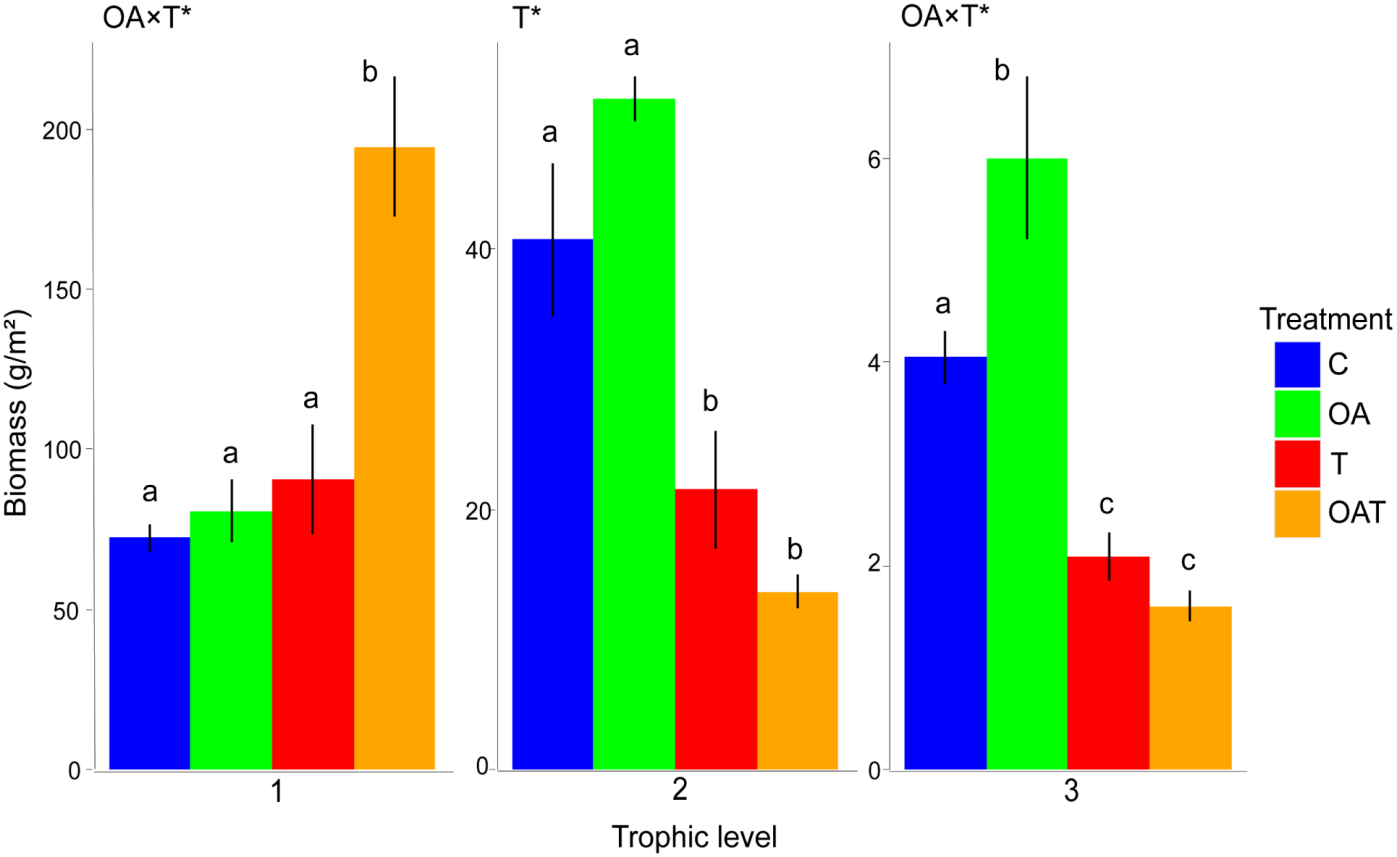
Living biomass of primary producers (trophic level 1), primary consumers (level 2), and secondary consumers (level 3) across functional groups within the mesocosms. The biomass of functional groups with intermediate trophic levels (e.g., trophic level of filter feeders = 2.4) was assigned to the levels 2 and 3 according to their relative contribution to trophic flow (e.g., 60% to level 2 and 40% to level 3). At the third trophic level, the decrease in biomass under T and OAT is primarily driven by filter feeders, while a negative effect was not apparent in most other functional groups such as the fishes (see S2 Fig). Living biomass includes 16 functional groups excluding detritus. Values are means ± SE across mesocosms (*n* = 3). Significant interactions or main effects (*p* < 0.05) within trophic levels are based on two-way ANOVAs (df = 1,8) and are indicated with asterisks. See S2 Table for statistical test outcomes. Means with different lowercase letters indicate significant difference among treatments based on posthoc tests corrected for false discovery rate and done separately for different trophic levels. C, control; OA, elevated CO_2_, OAT, elevated CO_2_ and temperature; T, elevated temperature.

**Fig 3:**
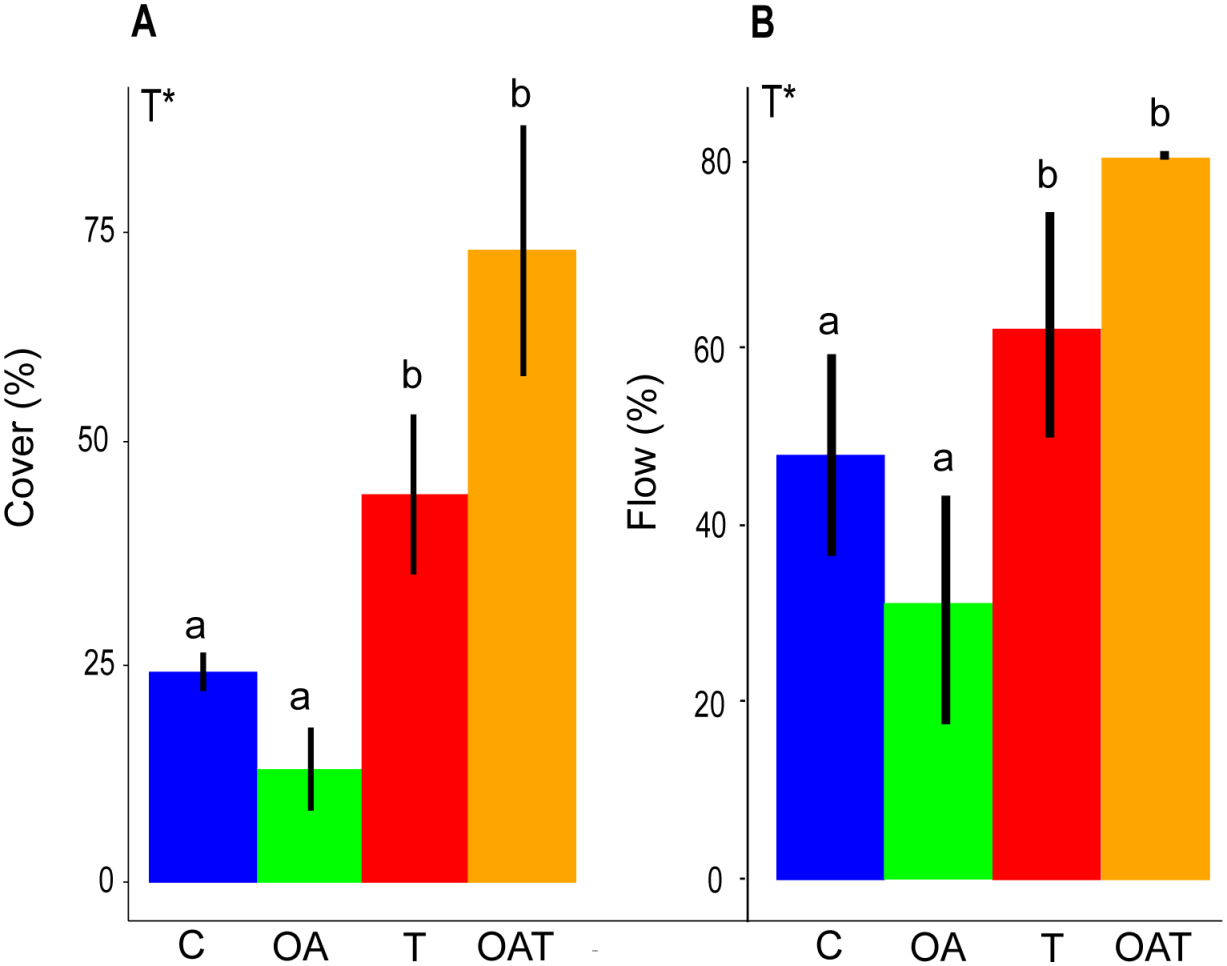
Relative proportion of cyanobacteria (as a percentage) to turf algae in mat-forming algae measured as benthic cover (A). Flows of production (%) to detritus pool relative to primary productivity (B). Mean ± SE values per mesocosm are given (*n* = 3). Significant main effects (*p* < 0.05) are based on two-way ANOVAs (df = 1,8) and are indicated with asterisks. Means with different lowercase letters indicate significant difference among treatments. See S3 Table for statistical test outcomes. C, control; OA, elevated CO_2_, OAT, elevated CO_2_ and temperature; T, elevated temperature.

Whilst the combined effect of warming and acidification enhanced the biomass of primary producers (*p* = 0.001; post hoc living biomass: *OAT* > control) and acidification enhanced secondary consumer biomass (*p* = 0.034; post hoc living biomass: *OA* > control), warming (*T* and *OAT*), irrespective of ocean acidification, caused a decrease in living biomass at trophic levels 2 and 3 (Fig 2; S2 Table). Warming (*T* and *OAT*) induced higher cyanobacterial biomass (ANOVA, F_1, 8_ = 19.90, *p* = 0.002), replacing palatable turf algae at trophic level 1 (Fig 3a, S3a Table). Consequently, energy was not transferred to successive trophic levels through consumption but accumulated as detrital biomass (ANOVA, F_1, 8_ = 9.12, *p* = 0.017) (Fig 3b; S3b Table) at the bottom of the food web. The system became less efficient in recycling the accumulated detrital biomass under warming (S3 Fig; S3c Table; ANOVA, F_1, 8_ = 9.31, *p* = 0.016), suggesting a collapse at the base of the food web.

## Discussion

Our study provides strong empirical evidence that global warming has the capacity to drive a collapse in marine food webs by altering energy flows between successive trophic levels. In our ecologically complex benthic mesocosm experiment, the combination of warming and acidification enhanced the biomass of primary producers, but reduced energy flow to herbivores, while warming (irrespective of acidification) reduced energy flow to carnivores at higher trophic levels. Warming also decreased the trophic transfer efficiency between primary producers and herbivores, consequently reducing standing biomass of herbivores and carnivores. Other studies based on the metabolic theory of ecology (MTE) [32], however, suggest that temperature-driven increased primary production is likely to propagate through food webs via strong top-down control [40,41], resulting in greater levels of heterotrophic biomass, relative to autotrophic biomass [4]. In our case, the combination of warming and acidification decoupled increased basal productivity from herbivore production, while warming in isolation reduced predator production, making most of the primary production unavailable further up the food chain. Thus, energy from enhanced primary producer biomass under future climate conditions may not always transfer through to successive trophic levels, but instead can decouple food demand and supply. Such a decoupling may alter dietary preferences of consumers, modifying consumer–prey relationships within food webs.

Our current inability to capture more realistic features of food web responses to global climate change is mostly due to a reliance on short-term, small-scale experiments harbouring single species and lower trophic levels, which provide an ambiguous approximation of naturally complex food webs [4]. Moreover, few studies of ecological responses to climate change include predator–prey dynamics and competitive interactions or allow for the potential proliferation of opportunistic groups of species, all of which can greatly influence or reverse many of the predicted responses of species and communities to climate change [26]. In contrast, our experimental results account for complex multispecies interactions and biotic processes, providing an improved representation of natural systems and how these are likely to respond to global warming [42,43]. Our experimental data provide insights into how anthropogenic climate change can potentially affect food web dynamics for relatively short-lived taxa. This is because large scale mesocosm experiments such as ours bridge the gap between simplified experimental conditions and the real world [44], providing important opportunities to better understand the likely mechanisms by which primary productivity under certain future climate conditions propagates through the food web.

Increased standing biomass in our benthic mesocosm experiment was most evident for cyanobacteria under warming (irrespective of acidification). Warming is known to enhance the primary productivity of some taxa, particularly of weedy species such as turf algae [45]. Cyanobacteria form a major component of turf algae [46,47] and are predicted to proliferate and expand under eutrophication and climate change [48]. The potential for cyanobacterial dominance in turf algal assemblages under future warming and acidification has been demonstrated previously using experimental approaches [49]. The effect of warming sea temperatures is expected to be greater in areas that are shallow and nutrient rich [50], providing ideal environmental conditions for cyanobacteria to invade other benthic primary producer groups [51,52]. Some cyanobacteria are known to be toxic and cause localized anoxia and mortality in marine organisms [48]. Cyanobacteria also produce potent allelochemicals that deter feeding and are difficult to control by grazers [53]. Herbivores like macroinvertebrates and small epifaunal invertebrates predominantly feed on mat-forming turf algae rather than cyanobacteria.

Metabolic theory suggests that consumers will show a greater response to temperature than producers [4,54,55]. Therefore, we hypothesised a priori that warming would drive an increase in metabolic and consumption rates for macroinvertebrates in our experiment. However, reduced food availability, brought about by palatable types of turf algae being replaced by unpalatable cyanobacteria, caused food limitation, preventing increased metabolic rates for macroinvertebrates at higher temperatures, suppressing the flow of energy to the second trophic level [16,56]. Furthermore, biomass of major prey groups such as copepods, small epifaunal invertebrates, and filter feeders, which largely form the diet of consumers at the third trophic level, collapsed under the warming treatments, resulting in significantly less energy available for the third trophic level. One of the reasons for this collapse is an excessive predation pressure on primary consumers by species at the third trophic level (i.e., predators) due to their higher energetic demand [28], which was not matched by any increase in productivity rates of primary consumers under warming. Alternatively, under warming, the consumer–producer relationship at the base of the food web could be nonsynchronous if consumption rates of herbivores peak earlier than the growth rates of producers, creating a mismatch between production and consumption [45]. This means that under certain conditions, even in the absence of herbivory-resistant primary producers, warming-induced metabolic stress of organisms can effectively decouple the consumer–producer relationship if consumer metabolism and consumption cannot keep pace with increasing production.

In our experiment, the collapse of biomass of major functional groups under warming played a major part in reducing the transfer efficiency of energy between trophic levels in a food web. A lower transfer efficiency of energy was evident between trophic levels 1 and 2. This is because transfer efficiency depends on both the availability of food, the biomass of all consumers, and their consumption rates. The lower standing biomass at trophic level 2 and reduced palatable food abundance at trophic level 1 collectively brought about lower overall transfer efficiency between these trophic levels under warming. Metabolic theory states that the structure and dynamics of ecological communities are based on the individual metabolism of organisms where the individual metabolic rate is primarily controlled by body size, body temperature, and resource availability [57]. Metabolic theory does not specifically consider the standing biomass of consumers, which is an important component of our model, allowing us to calculate transfer efficiency. Therefore, food web properties, such as transfer efficiency, are difficult to interpret from the perspective of metabolic theory, yet they can have important community-level effects [58].

The replacement of turf algae by cyanobacteria further resulted in a more detritus-based food web under warming. Detritus can be very important for sustaining food webs and ecosystem stability [59], but only when proper recycling occurs within the system [60]. Decreasing detritus recycling in ecosystems correlates with decreased system resilience to perturbations, with lower rates of recycling resulting in slower recovery [60,61]. We show (based on the Finn's cycling index) that warming significantly reduced the detritus recycling capacity of the system. The inability of herbivores to consume enhanced primary producer biomass and the simultaneous failure of detritivores to transfer excessive detritus production to the successive trophic levels resulted in an accumulation of detrital biomass at the base of the food chain. Therefore, warming-induced detritus accumulation, as observed in our study, might have far-reaching ecosystem consequences for future oceans, such as the spreading of ‘dead zones’ through increased microbial activity and consumption of dissolved oxygen in bottom waters [30].

Reduced secondary and tertiary production has been forecast under future acidification [2]. However, this pattern was not detected in our experiment in energy flows, a proxy for production at different trophic levels. We show that acidification can in fact exert positive bottom-up effects on energy flow towards secondary producers (trophic level 2). Ocean acidification could increase secondary productivity in situations where strong indirect positive effects dampen the direct negative effects of elevated CO_2_, i.e., through increased habitat and food, as well as reduced predator abundances [62,63]. Only benthic carnivores and carnivorous fish at trophic level 3 experienced increased energy flows under acidification, which supports the finding of increased productivity of a carnivorous fish (*Favongobius lateralis*) in a previous study done in a similar ecological setting [28]. In contrast, omnivorous fish showed a decrease in flow and this group was the major contributor of energy flow to trophic level 3 under control conditions. Taken together, the positive effect of acidification on the energy flows of some functional groups and negative or lack of effects on other groups resulted in no overall significant increase in energy flow to trophic level 3 under acidification. Here we quantify secondary and tertiary production, using a more complex (and ecologically realistic) food web, which better captured real-world community structure and important species interactions, and how these are likely to change in response to future global warming and ocean acidification [8,39,64]. This broader food web model also allows us to quantify energy transfer efficiency across multiple trophic levels; an important ecosystem function which can regulate many ecological processes (i.e., trophic structure, food chain length) and mediate ecosystem services (i.e., fisheries production) [65–67]. Our results indicate that the response of future food webs to ocean acidification and temperature are likely to depend on the localized community composition and consumer–resource interactions of the specific ecosystem.

Although we used one of the most complex benthic mesocosm experiments to date, our approach is not devoid of caveats. For example, difficulties in separating the functional roles of turf and cyanobacteria meant that they were modelled as one functional group ‘mat-forming algae’. In our model, we did not consider regular bacteria (other than cyanobacteria) as a separate functional group, but rather considered them under detritus. Thus, detritivores are considered to mainly feed on detritus and its associated bacteria. We opted for not using an extra bacterial compartment because bacteria would largely overshadow any other trophic flows of the system [68]. Our study showed a relatively larger biomass flux between trophic levels 1 to 2 compared to between trophic levels 2 and 3 due to the presence of relatively large-bodied primary consumers (such as herbivorous macroinvertebrates: *Bulla quoyii*), which were too big to handle for the gape-limited predators in our system. The presence of a wider range of higher-order invertebrate predators, as is the case in natural ecosystems, would have reduced this disproportionally high flux between primary producers and primary consumers by stronger top-down control. However, since the focus of our study was to show relative difference among the climate treatments, the results still provide a valuable quantitative insight into the potential future of some benthic marine ecosystem under two co-occurring global climate stressors.

One of the weaknesses of earlier applications of the Ecopath model were assumptions of ‘steady-state’ or equilibrium conditions, meaning that the model outputs should only be considered for the period across which the model input parameters are deemed valid [69]. Ecopath modelling approaches now no longer assume steady-state conditions but instead the model parameterizations are based on a mass-balance assumption over a chosen arbitrary period. The mass-balance approach in Ecopath filters for mutually incompatible estimates of flow [69]. Moreover, under the Ecopath modelling approach, we assumed that mortality for a prey equals consumption of a predator and that all prey are equal in terms of energetic content. Additional care needs to be taken when inferences are drawn from ecosystem models built for highly dynamic systems due to likely nonlinearities in important food web properties of some functional groups, operating at fine temporal scales. Nevertheless, since we used multiple sampling points through time and averages based on multiple replicates for our model input, especially for taxonomic groups that have the potential to show large oscillations with environmental fluctuations, the model outputs are likely to be indicative of near-future ecological states. Lastly, model outputs were tested using the PREBAL diagnostic and pedigree index (see Supplementary Methods for details on model and data quality), and confirmed a stable model that is ecologically robust.

In summary, our results suggest that warming has the capacity to drive an energetic collapse at the base of marine food webs, and this effect can propagate to higher trophic levels—subsequently leading to a collapse in species biomass of the entire food web. The underlying mechanism for this collapse is the replacement of preferred turf algae by cyanobacterial biomass that drives the system towards food limitation for herbivores, with detrimental effects on their predators, combined with a switch towards a less efficient detritus-driven system. Several studies have reported an apparent increase in the occurrence of cyanobacteria in marine waters globally as a result of increasing temperatures [52], and regionally in temperate [70], tropical [51,71], and subtropical [72,73] areas. Thus, these findings are particularly important in the context of climate change, as mismatches in trophic dynamics can decouple linkages between trophic levels driving ecosystems towards simplified, less productive systems, with cascading effects on ecosystem resilience and functioning.

## Materials and methods

### Ethics statement

This research was carried out under the approval of the University of Adelaide animal ethics committee (approval S-2012-193A). All the habitats and organisms collections were permitted by the Minister for Transport and Infrastructure and the Government Department of Primary Industry and Regions SA (exemptions: 9902676 and 9902752).

### Experimental design

An indoor mesocosm experiment was maintained from February 2015 to July 2015. A total of 12 circular mesocosms, each holding 1,800 L of water were set up inside a large temperature-controlled room to simulate shallow temperate coastal ecosystems typical of the Gulf St. Vincent, South Australia (S4 Fig). All habitats and organisms used in the mesocosms were collected at a depth of 1–5 m within 60 km distance of the mesocosm facility. Each mesocosm comprised of a mosaic of the 3 primary local habitats (with 2 replicate patches per habitat per mesocosm): rocky reef, seagrass, and open sand [74].

Rocky reefs consisted of natural rocks collected in situ and included attached macrophytes dominated by an assemblage of fucoids (Order Fucales; mainly species belonging to the families Fucaceae and Sargassaceae) and benthic invertebrates. Rocks were selected to be as similar as possible in terms of presence and cover of major fucoid species. Seagrass habitat was mimicked by artificial green polypropylene ribbon harbouring epiphytes and planted into fine silica sand at a depth of 6 cm. The seagrass habitat resembled the most abundant local seagrass *Posidonia* spp. [74] and was incubated in situ for 2 weeks to allow for epiphytic colonization. The circular ‘rocky reef' patches and ‘artificial seagrass’ patches were of equal size (0.42 m diameter). The space in between and around these patches was ‘open sand’ habitat, comprising fine silica sand with a depth range between 6–25 cm. The open sand and sand within the seagrass patches were additionally seeded with 0.025 m^3^ natural sediment collected in situ between patches of live seagrass and included all infauna and flora.

Fish and invertebrates were introduced into the mesocosm and represented different feeding guilds (see S6 Table for a list of species associated with the mesocosms, their stocking density, and mean sizes). The fishes were selected based on their high local juvenile abundances in shallow coastal waters during summer, while the gastropods came from the rocks used to build the rocky reef patches and were redistributed evenly among all mesocosms. In the flow-through system, unfiltered seawater from 1.5 km off-shore and 8 m depth continuously supplied nutrients and planktonic propagules to each mesocosm at 2,300 L day^−1^. A diffuser was used to form a light circular current in the mesocosms to simulate tidal water movement alternating direction in 6-hour intervals (S4 Fig). A lamp was mounted above each mesocosm with a spectrum close to sunlight, which is roughly equivalent to 72.83 ± 24.78 μmoles/m^2^/second Photon flux corresponding to a local water depth of 6–7 m (14/10 light-dark cycle, 30-minute dawn and dusk dimming).

We applied a control temperature of 21.0°C in our mesocosm experiment corresponding to the average summer temperature based on a 5-year dataset of 2 local temperature loggers (5 m depth, 2010–2015, SA Water). *OA* at each mesocosm was achieved through a header tank preconditioned to elevated *p*CO_2_ levels using pure CO_2_ (control system ACQ110 Aquatronica, Italy). Additionally, each mesocosm was supported by a 60-L bin bubbled heavily with enriched air at 1,000 ppm *p*CO_2_ (PEGAS 4000 MF Gas Mixer, Columbus Instruments, Columbus, Ohio) or ambient air at 400 ppm *p*CO_2_, to maintain target levels. Submersible titanium heaters inside the 60-L bins were used for the future warming treatments. Temperature and pH were measured daily (Mettler Toledo SevenGo SG2, calibrated daily; S5 Fig), while salinity was measured fortnightly (SR6 refractometer; Vital Sine). The total alkalinity was also measured fortnightly by Gran titration from water samples (888 Titrando, Metrohm, Switzerland). The diurnal variability in pH (S6 Fig) confirms that our mesocosms were autonomous systems that mimicked natural day-night fluctuations. For a description of other seawater properties, see S7 Table.

### Species sampling and processing

The fish community, herbivorous macroinvertebrates, small epifaunal invertebrates, filter feeders, and macro-crustaceans were all sampled and counted at the end of the experiment and their biomass measured as wet weight.

Biomass of tanaids, copepods, and meiobenthos was determined using benthic samplers (6.5 cm in diameter and 2 cm depth filled with 1.5 cm of mesocosm sand, with 2 replicate samplers per mesocosm), which were placed at the bottom of the tanks for about a month, allowing colonization of these species. Samples were collected twice from each mesocosm during the experimental period and pooled for each mesocosm prior to processing. Tanaids, meiobenthos, and copepods were extracted from the sand within the benthic samplers via floatation using a Ludox TM colloidal solution with a specific gravity of 1.18. The animals were then collected using a 120 μm sieve. Microzooplankton biomass was measured following volumetric method [75] by filtering 400 L of water from each mesocosm through a plankton sampler at the end of the experiment.

Phytobenthos biomass was measured using the same benthic samplers as above. Two samplers were placed in each mesocosm for biomass measurements. A micro-spatula was used to carefully scrape the thin phytobenthic layer from the upper surface (approximately 1 mm thick) of the sand. The remaining sand was filtered through a pre-combusted and pre-weighed Whatman GF/C glass fiber filters to determine the detritus biomass. In the laboratory, photosynthetic pigments were extracted from freeze-dried sand samples (0.3–0.6 g) with 10 ml 90% acetone. After 48 hours of darkness at –20°C, the samples were stirred in a vortex, centrifuged at 3,500 rpm for 15 minutes, and extracts were analyzed in a 6,405 UV/Vis, Jenway spectrophotometer and their concentration calculated [76].

Phytoplankton biomass (measured as Chlorophyll *a*) was quantified based on photosynthetic pigment concentration. Four litres of water were filtered from each mesocosm with Whatman GF/C glass fiber filters of 4.7 cm diameter, and ground and extracted [76]. Samples for both phytobenthos and phytoplankton were collected twice during the experimental period, and the average of both was used as the model input.

To estimate the biomass of macrophytes and mat-forming algae, all habitats (rock, seagrass, and open sand) were sampled at the end of the study period. Their wet weight was determined to the nearest 0.1 mg. Mat-forming algae were defined as a mix of turf and cyanobacteria. The relative cover of cyanobacteria in mat-forming algae was estimated using the Coral Point Count with Excel Extensions (CPCe) Software [77]. In addition, community metabolism was measured as gross oxygen production (mgO_2_/m^3^/min^1^) once per mesocosm at the end of the study. Oxygen concentration was measured in 1 minute intervals over at least 30 minutes (HQ40d Portable Meter, sensor LDO101, HachTM). A linear regression model of O_2_ production rate (where O_2_ concentration was modelled as function of time) was fitted and confirmed a high level of precision in the measurement of O_2_ concentrations across the 12 mesocosms (mean ± SD, R^2^ = 0.94 ± 0.04).

### Food web model construction

We built mass-balanced food web models. Trophic links were weighted by material fluxes among functional groups using Ecopath [78]. We modelled energy or mass flow over a 4 month time step based on local summer conditions today and in the future. We then converted and expressed the model produced energy flow results from the experimental period to values/month to make it more comparable to other systems.

Our model used the following input parameters: biomass, represented by the value B; production per unit of biomass, represented by the value P/B; consumption per unit of biomass, represented by the value Q/B; diet matrix; and the model-estimated ecotrophic efficiency, represented by the value EE. The latter is a parameter that is derived from the model, describing the fraction of the productivity that is used in the system. Most of these model parameters were calculated using our empirical mesocosm data, including final biomass (end-of-experiment) and diet composition of consumers of various functional groups (see S8, S9 and S10 Tables). The Q/B ratio for most of the functional groups was calculated using stomach content analysis and in situ feeding trials that incorporated treatment effects. Therefore, it incorporates the direct effects of temperature on metabolism, accounting for estimates of trophic biomass fluxes and efficiencies. In cases where data were not available, data were derived from empirical equations and published information (see S1 Text).

Flow and transfer efficiency were based on the trophic aggregation routine [69] that aggregates the entire system into discrete trophic levels sensu [79]. The discrete trophic levels start with level I, corresponding to the primary producers and the nonliving, detrital compartments. Strict herbivores or detritivores consequently occupy a position of level II. This is followed by higher-order consumers that are allocated to several discrete trophic positions according to the type and amounts of food that reach them along feeding pathways.

Energy flows were calculated for different trophic levels following [80], where—for example—if a group obtains 40% of its food as an herbivore and 60% as a carnivore, the corresponding fractions of the flow through the group are attributed to the herbivore level and the carnivore level, respectively. The relative flows (these are proportions adding up to 1) were converted to absolute amounts and shown as the net amount of energy that flows to higher trophic levels through consumption (g/m^2^/month). The ‘transfer efficiency’ is the percentage of trophic flows at trophic level *n* that is converted into flows at level *n + 1*. The transfer efficiency of a given trophic level (trophic level = *n*) not only depends on the available energy at a given trophic level (trophic level = n) but also the standing biomass at the next trophic level (trophic level = *n + 1*). The transfer efficiencies between successive discrete trophic levels were calculated as the sum of the flow that is transferred from any given level to the next higher level, plus exports from the original level relative to the throughput (or input) of the given (originating) trophic level [69,78]. The throughput is the sum of all flows (such as consumption, export, respiration, and flows to detritus) in a given trophic level and represents the ‘size of the trophic level in terms of flow’ [81].

Calculation of transfer efficiency from trophic level 1 to 2 is not possible without having information on gross primary production or respiration [82]. We measured net productivity and respiration in each mesocosm and used them to estimate the transfer efficiency between trophic level 1 and 2 for each mesocosm food web [69,78]. The initial output for both energy flow and transfer efficiency was obtained for discrete trophic levels I to V. However, for the simplicity of the model output and better visualization, we pooled the data for trophic levels III to Vand showed this as 1 integrated trophic level (i.e., trophic level 3).

We used the Ecopath pedigree routine to quantify the uncertainty associated with the model parameters by recording the origin and quality of the input data and assigning a value of uncertainty or a confidence interval to each input (e.g., biomass, P/B, Q/B, diets), which are then used to calculate the overall model pedigree index. The pedigree index varies between 0 (low precision information) and 1 (high quality, i.e., obtained from modelled system and highly precise), allowing a description of the quality of the model [78]. Our overall individual model pedigree index of 0.71, and a measure of fit of *t* = 3.819, indicates a very high quality and robust model compared to 393 previously constructed models from habitats from around the world, for which pedigree values ranged between 0.164 and 0.676 [83]. More details on the parameterization and model computation are given in the Supplementary Methods.

### Statistical analysis

The effects of warming and ocean acidification on food web properties (response variables: absolute energy flow, transfer efficiency, and standing biomass) were analysed using two-way ANOVAs. Both climate factors were treated as fixed and orthogonal. Before analysis, normality was checked for all response variables using the Shapiro-Wilk test, and homogeneity of variances was assessed using a Levene’s test as well as by evaluating plots of residuals against predicted values. Response variables were log_10_ transformed prior to analysis if they did not conform to a normal distribution. For significant interactions, posthoc multiple comparisons adjusted by false discovery rate were performed [84]. All data analyses were done with the software package R version 3.2.3 [85].

## Supporting information

S1 Fig Absolute flows (gWWm^−2^month^−1^) produced by the different functional group at trophic level 2. Mean ± SE values per mesocosm are given (*n* = 3). Significant interactions or main effects (*p* < 0.05) within functional groups are based on two-way ANOVAs (df = 1,8) and are indicated with asterisks. Means with different lowercase letters indicate significant difference among treatments based on posthoc tests corrected for false discovery rate and done separately for different functional group. No Sig = no significance. See S4 Table for statistical test outcomes.
(TIF)

S2 Fig Absolute flows (gWWm^−2^month^−1^) produced by the different functional group at trophic level 3. Mean ± SE values per mesocosm are given (*n* = 3). Significant interactions or main effects (*p* < 0.05) within functional groups are based on two-way ANOVAs (df = 1,8) and are indicated with asterisks. Means with different lowercase letters indicate significant difference among treatments based on posthoc tests corrected for false discovery rate and done separately for different functional groups. No Sig = no significance. See S5 Table for statistical test outcomes.
(TIF)

S3 Fig The Finn’s cycling index expresses the amount of detritus that is recycled relative to the total throughput of the system. Mean ± SE values per mesocosm are given (*n* = 3). Significant effects (*p* < 0.05) are based on two-way ANOVAs with *OA* and *T* (df = 1,8) and are indicated with asterisks. Means with different lower case letters indicate significant difference among treatments. See S3 Table for statistical test outcomes. *OA*, elevated CO_2_; *T*, elevated temperature.
(TIF)

S4 Fig The different structural components of the mesocosm used for this experiment. Each mesocosm comprises 4 ‘rocky reef’ patches (A) and 4 ‘artificial seagrass’ patches (B). The space in between and around these patches was considered ‘open sand’ habitat (C). The incoming seawater was led into 2 header tanks (800 L) at the beginning of the flow-through facilities, and from there gravity-fed into each mesocosm (D). The header tank was preconditioned to future *p*CO_2_ levels with pure CO_2_ (control system ACQ110 Aquatronica, Italy) prior to supplying the water to the 6 acidified mesocosms. In addition, continuous water circulation (approximately 1,800 L per hour) was maintained between each mesocosm and a 60-L supporting bin positioned next to each mesocosm that was bubbled heavily with enriched air at 1,000 ppm *p*CO_2_ (PEGAS 4000 MF Gas Mixer, Columbus Instruments, Columbus, Ohio) or ambient air at 400 ppm *p*CO_2_, depending on the acidification treatment. These bins also contained the submersible titanium heaters for the *T* treatments. A diffuser pipe was used to generate a mild circular current inside the mesocosms using the water exchange between supporting bin and mesocosm and alternating direction every 6 hours (E). A filter column (approximately 20 μm) allowed water to flow back into the 60-L bin by gravity (F) and ensured that larger organisms were always retained within the mesocosms. In summary, this technically complex set-up ensured a mesocosm environment without unnatural disturbances such as pump noise, air bubbles, or electrical currents. A 250W metal halide lamp (Osram Powerstar HQI-T 250/D/PRO) mounted just above the mesocosm (G) ensured an irradiance that corresponded to approximately 6–7 m water depth in Gulf St. Vincent (Phillips et al. 1981). *T*, elevated temperature.
(TIF)

S5 Fig Variability in pH and temperature over the 5-month study period. This includes 3 phases: 1) the first week of the acclimation period, 2) the progressive elevation to treatment levels, and 3) at maintained treatment levels. Mean ± SD are shown based on 3 mesocosms for each treatment. pH and temperature were both measured once daily in each mesocosm around midday.
(TIF)

S6 Fig Diurnal variability in pH measured over a 5-day period in the middle of the study period. This analysis was only done for 1 mesocosm per treatment combination, serving as an example. For these 4 mesocosms in parallel, pH was recorded at 30-minute intervals with an automated pH logger (control system ACQ110 Aquatronica, Italy).
(TIF)

S7 Fig Schematic diagram showing the different phases of model building and execution. A) data collection from the mesocosms and parameter estimation, B) mass-balance modelling in Ecopath, and C) model balancing and validation.
(TIF)

S8 Fig PREBAL of the control and acidification models plotting (a) biomass estimates (gWWm^−2^), (b) production/biomass ratio (per 4 months), and (c) consumption/biomass (per 4 months) on a log scale versus functional groups ranked by their trophic level, from lowest to highest trophic level. A constant of 1 was added to all response variables to avoid some negative values (Log_10_ [*x* + 1]) prior to PREBAL plotting. For specific functional group name, refer to the legend. Herb. = herbivorous. PREBAL is shown only for base models that are built on the average of all the input parameters (B, P/B, Q/B) across mesocosms within each climate treatment. B, biomass; P/B, production per unit of biomass; Q/B, consumption per unit of biomass.
(TIF)

S9 Fig PREBAL of the temperature and temperature + acidification models plotting (a) biomass estimates (gWWm^−2^), (b) production/biomass ratio (per 4 months), and (c) consumption/biomass (per 4 months) on a log scale versus functional groups ranked by their trophic level, from lowest to highest trophic level. A constant of 1 was added to all response variables to avoid some negative values (Log_10_ [*x* + 1]) prior to PREBAL plotting. For specific functional group name, refer to the legend. Herb. = herbivorous. PREBAL is shown only for base models that are built on the average of all the input parameters (B, P/B, Q/B) across mesocosms within each climate treatment. B, biomass; P/B, production per unit of biomass; Q/B, consumption per unit of biomass.
(TIF)

S1 Table Analysis of variance of the effects of *OA* and *T* and their interaction on absolute flows and transfer efficiency between successive trophic levels of the food web. Significant differences are indicated with asterisks, with *: *p* < 0.05, **: *p* < 0.01, and ***: *p* < 0.001. *OA*, elevated CO_2_; *T*, elevated temperature.
(XLSX)

S2 Table Analysis of variance of the effects of OA and *T* and their interaction on living biomass by trophic levels of the food web. Significant differences are indicated with asterisks, with *: *p* < 0.05, **: *p* < 0.01, and ***: *p* < 0.001. *OA*, elevated CO_2_; *T*, elevated temperature.
(XLSX)

S3 Table Analysis of variance of the effects of *OA* and *T* and their interaction on cyanobacteria (% cover), flow (%) to detritus, and Finn’s cycling index in the food web. Significant differences are indicated with asterisks, with *: *p* < 0.05, **: *p* < 0.01, and ***: *p* < 0.001. *OA*, elevated CO_2_; *T*, elevated temperature.
(XLSX)

S4 Table Analysis of variance of the effects of *OA* and *T* and their interaction on the absolute flows of contributing functional groups from trophic level 1 to 2. Functional groups were ordered in terms of their contribution to total energy flows. Significant differences indicated with asterisks, with *: *p* < 0.05, **: *p* < 0.01, and ***: *p* < 0.001. *OA*, elevated CO_2_; *T*, elevated temperature.
(XLSX)

S5 Table Analysis of variance of the effects of *OA* and *T* and their interaction on the absolute flows of contributing functional groups from trophic levels 2 to 3. Functional groups were ordered in terms of their contribution to total energy flows. Significant differences are indicated with asterisks, with *: *p* < 0.05, **: *p* < 0.01, and ***: *p* < 0.001. *OA*, elevated CO_2_; *T*, elevated temperature.
(XLSX)

S6 Table List of species/taxa and their respective functional group considered in the mesocosm food webs. (XLSX)

S7 Table Mean (± SD) seawater parameters in the experimental mesocosoms with 2 crossed factors of *T* and *OA*. Standard deviations represent the variability between individual mesocosms. *OA*, elevated CO_2_; *T*, elevated temperature.
(XLSX)

S8 Table Input (nonitalic) and output (italic) parameters for the ecosystem components used in C models. TL: trophic level, B: biomass (gWWm^−2^), P/B: production/biomass ratio (per 4 months), Q/B: consumption/biomass ratio (per 4 months), EE: ecotrophic efficiency, P/Q: gross food conversion efficiency of each functional group. CM1 represents control model 1, CM2 represents control model 2, and CM3 represents control model 3. The value in bold indicates that particular parameter varied among the models and mentioned below the table. C, control.
(XLSX)

S9 Table Input (nonitalic) and output (italic) parameters for the ecosystem components used in the *OA* models. TL: trophic level, B: biomass (gWWm^−2^), P/B: production/biomass ratio (per 4 months), Q/B: consumption/biomass ratio (per 4 months), EE: ecotrophic efficiency, P/Q: gross food conversion efficiency of each functional group. OAM1 represents acidification model 1, OAM2 represents acidification model 2, and OAM3 represents acidification model 3. The value in bold indicates that particular parameter varies among the models as mentioned below the table. *OA*, elevated CO_2_.
(XLSX)

S10 Table Input (nonitalic) and output (italic) parameters for the ecosystem components used in the *T* models. TL: trophic level, B: biomass (gWWm^−2^), P/B: production/biomass ratio (per 4 months), Q/B: consumption/biomass ratio (per 4 months), EE: ecotrophic efficiency, P/Q: gross food conversion efficiency of each functional group. TM1 represents temperature model 1, TM2 represents temperature model 2, and TM3 represents temperature model 3. The values in bold indicate that particular parameters vary among the models as mentioned below the table. *T*, elevated temperature.
(XLSX)

S11 Table Input (nonitalic) and output (italic) parameters for the ecosystem components used in the *OAT* models. TL: trophic level, B: biomass (gWWm^−2^), P/B: production/biomass ratio (per 4 months), Q/B: consumption/biomass ratio (per 4 months), EE: ecotrophic efficiency, P/Q: gross food conversion efficiency of each functional group. OATM1 represents temperature and acidification model 1, OATM2 represents temperature and acidification model 2, and OATM3 represents temperature and acidification model 3. The value in bold indicates that particular parameter varies among the models as mentioned below the table. *OAT*, elevated CO_2_ and temperature.
(XLSX)

S12 Table Predator/prey matrix (column/row) for C models. The fraction of one compartment consumed by another is expressed as the fraction of the total diet, the sum of each column being equal to 1. Values with mean ± SD represent the adjustment of different prey groups in predator diets across models. C, control.
(XLSX)

S13 Table Predator/prey matrix (column/raw) for *OA* models. The fraction of one compartment consumed by another is expressed as the fraction of the total diet, the sum of each column being equal to 1. Values with mean ± SD represent the adjustment of different prey groups in predator diets across models. *OA*, elevated CO_2_.
(XLSX)

S14 Table Predator/prey matrix (column/raw) for *T* models. The fraction of one compartment consumed by another is expressed as the fraction of the total diet, the sum of each column being equal to 1. Values with mean ± SD represent the adjustment of different prey groups in predator diets across models. *T*, elevated temperature.
(XLSX)

S15 Table Predator/prey matrix (column/raw) for *OAT* models. The fraction of one compartment consumed by another is expressed as the fraction of the total diet, the sum of each column being equal to 1. Values with mean ± SD represent the adjustment of different prey groups in predator diets across models. *OAT*, elevated CO_2_ and temperature.
(XLSX)

S16 Table Source of additional information used (√) to parameterize base (control model) models for different functional groups, where similar values used across treatments were specified in the supplementary text. (XLSX)

S1 Text Supplementary methods. (DOCX)

S1 Data Data used to generate the manuscript figures. (XLSX)

## Abbreviations

MTE: metabolic theory of ecology
*OA*: elevated CO_2_
*OAT*: elevated CO_2_ and temperature
*T*: elevated temperature
