# Supplementary figures and images for: Climate change could drive marine food web collapse through altered trophic flows and cyanobacterial proliferation
Source: PLoS Biol. 2018 Jan 9;16(1):e2003446. doi: 10.1371/journal.pbio.2003446 (PMC5760012; doi:10.1371/journal.pbio.2003446)

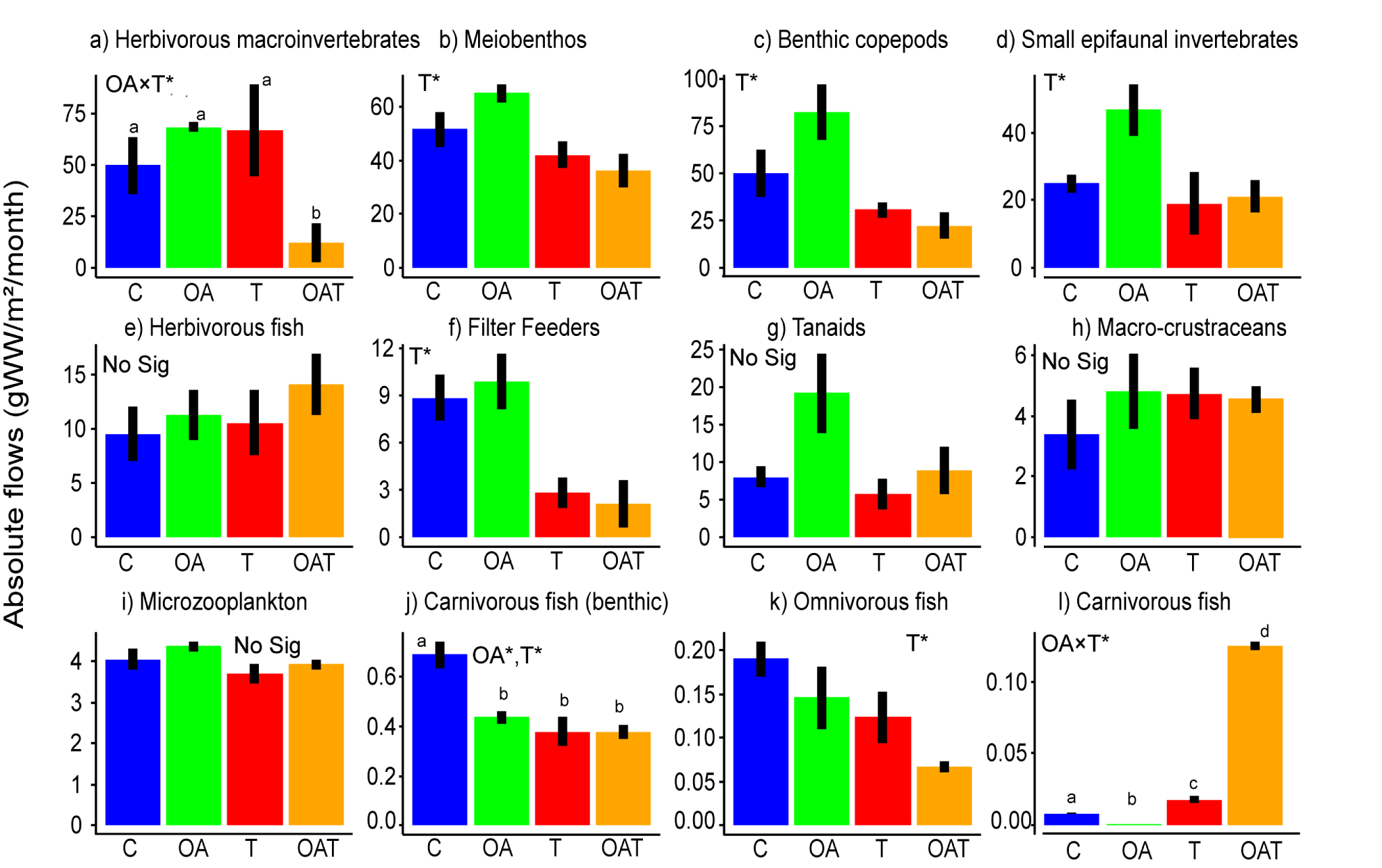

Supplement: S1 Fig — Mean ± SE values per mesocosm are given (n = 3). Significant interactions or main effects (p < 0.05) within functional groups are based on two-way ANOVAs (df = 1,8) and are indicated with asterisks. Means with different lowercase letters indicate significant difference among treatments based on posthoc tests corrected for false discovery rate and done separately for different functional group. No Sig = no significance. See S4 Table for statistical test outcomes. (TIF) [file pbio.2003446.s001.tif]

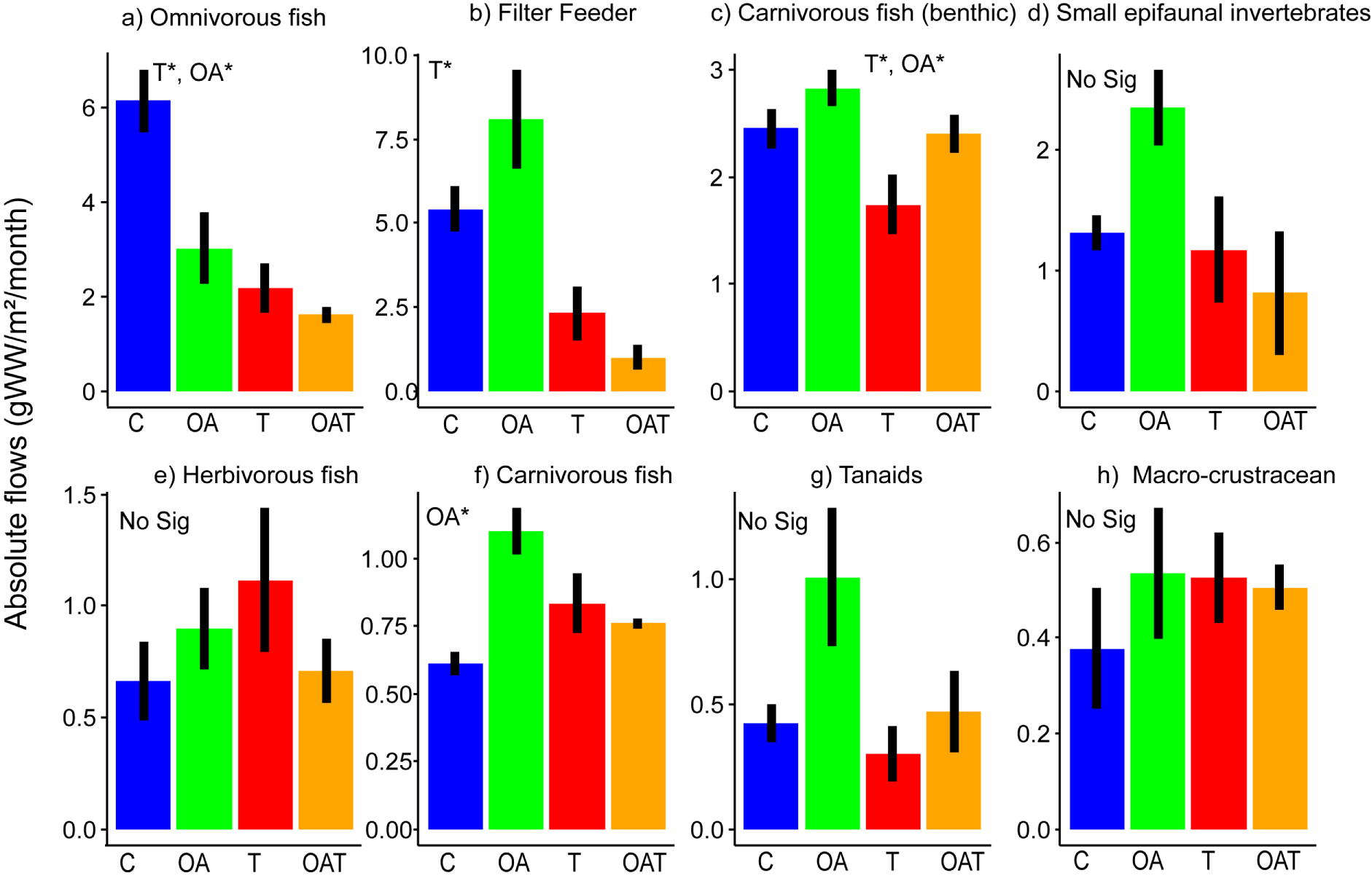

Supplement: S2 Fig — Mean ± SE values per mesocosm are given (n = 3). Significant interactions or main effects (p < 0.05) within functional groups are based on two-way ANOVAs (df = 1,8) and are indicated with asterisks. Means with different lowercase letters indicate significant difference among treatments based on posthoc tests corrected for false discovery rate and done separately for different functional groups. No Sig = no significance. See S5 Table for statistical test outcomes. (TIF) [file pbio.2003446.s002.tif]

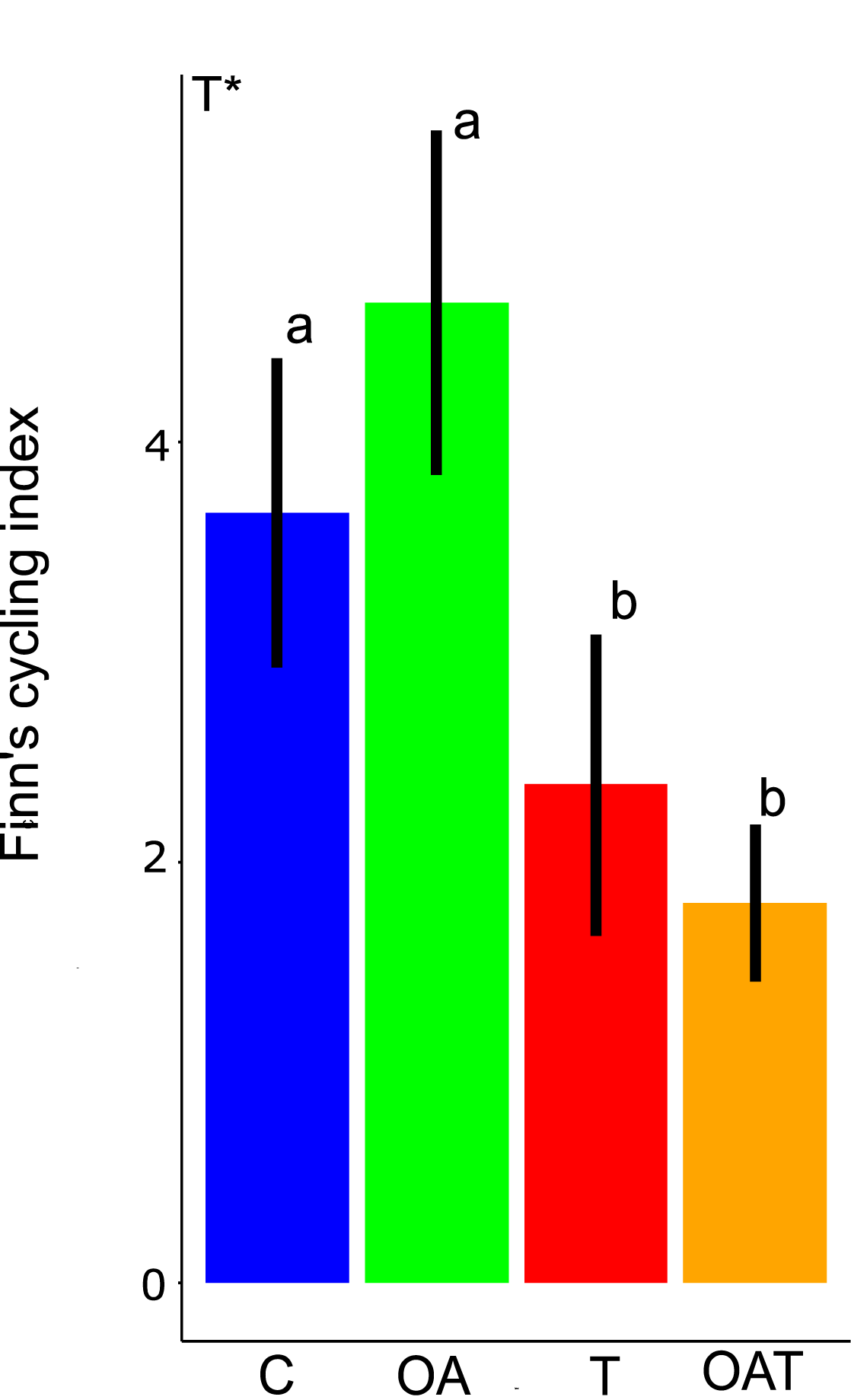

Supplement: S3 Fig — Mean ± SE values per mesocosm are given (n = 3). Significant effects (p < 0.05) are based on two-way ANOVAs with OA and T (df = 1,8) and are indicated with asterisks. Means with different lower case letters indicate significant difference among treatments. See S3 Table for statistical test outcomes. OA, elevated CO2; T, elevated temperature. (TIF) [file pbio.2003446.s003.tif]

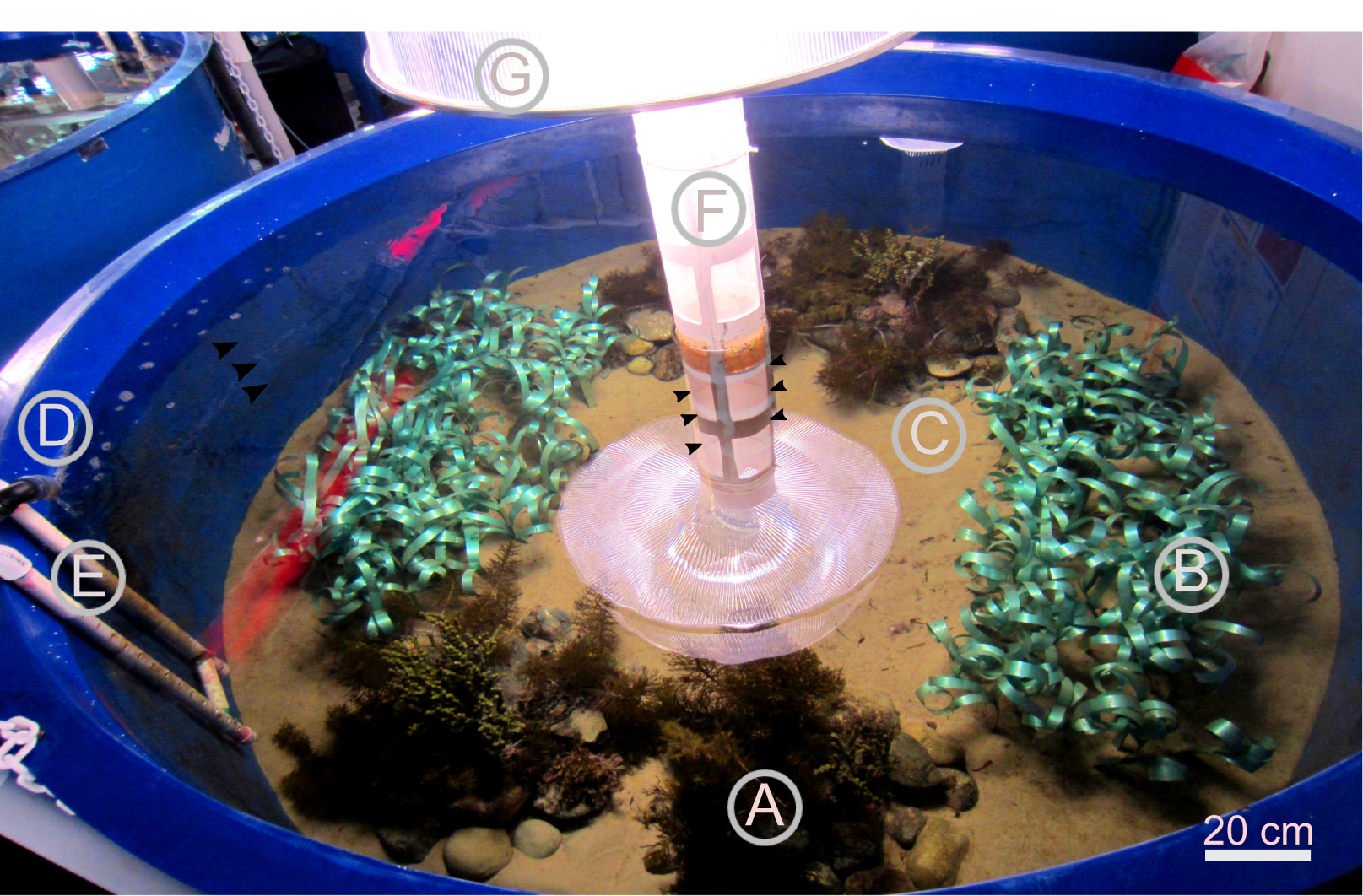

Supplement: S4 Fig — Each mesocosm comprises 4 ‘rocky reef’ patches (A) and 4 ‘artificial seagrass’ patches (B). The space in between and around these patches was considered ‘open sand’ habitat (C). The incoming seawater was led into 2 header tanks (800 L) at the beginning of the flow-through facilities, and from there gravity-fed into each mesocosm (D). The header tank was preconditioned to future pCO2 levels with pure CO2 (control system ACQ110 Aquatronica, Italy) prior to supplying the water to the 6 acidified mesocosms. In addition, continuous water circulation (approximately 1,800 L per hour) was maintained between each mesocosm and a 60-L supporting bin positioned next to each mesocosm that was bubbled heavily with enriched air at 1,000 ppm pCO2 (PEGAS 4000 MF Gas Mixer, Columbus Instruments, Columbus, Ohio) or ambient air at 400 ppm pCO2, depending on the acidification treatment. These bins also contained the submersible titanium heaters for the T treatments. A diffuser pipe was used to generate a mild circular current inside the mesocosms using the water exchange between supporting bin and mesocosm and alternating direction every 6 hours (E). A filter column (approximately 20 μm) allowed water to flow back into the 60-L bin by gravity (F) and ensured that larger organisms were always retained within the mesocosms. In summary, this technically complex set-up ensured a mesocosm environment without unnatural disturbances such as pump noise, air bubbles, or electrical currents. A 250W metal halide lamp (Osram Powerstar HQI-T 250/D/PRO) mounted just above the mesocosm (G) ensured an irradiance that corresponded to approximately 6–7 m water depth in Gulf St. Vincent (Phillips et al. 1981). T, elevated temperature. (TIF) [file pbio.2003446.s004.tif]

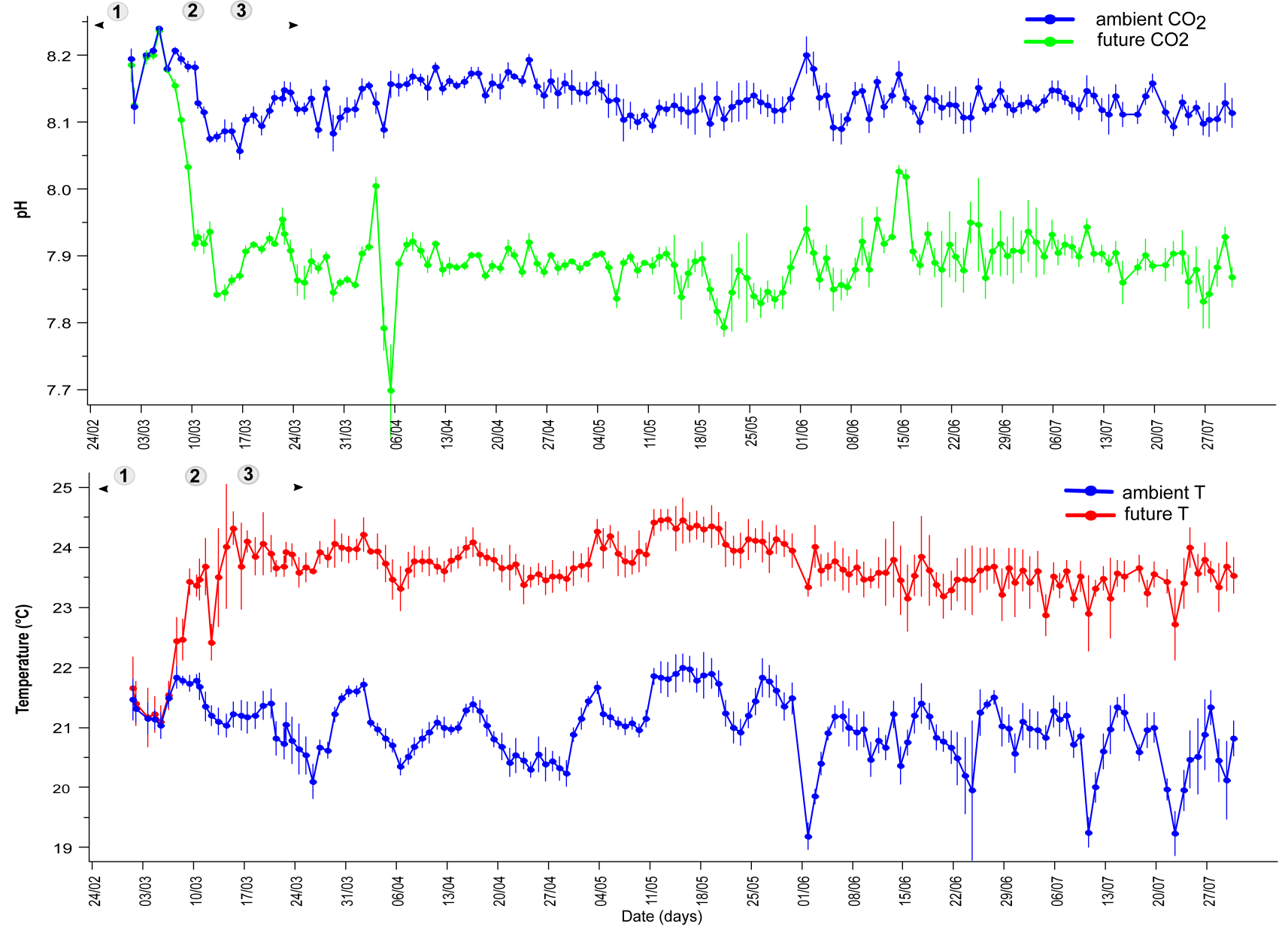

Supplement: S5 Fig — This includes 3 phases: 1) the first week of the acclimation period, 2) the progressive elevation to treatment levels, and 3) at maintained treatment levels. Mean ± SD are shown based on 3 mesocosms for each treatment. pH and temperature were both measured once daily in each mesocosm around midday. (TIF) [file pbio.2003446.s005.tif]

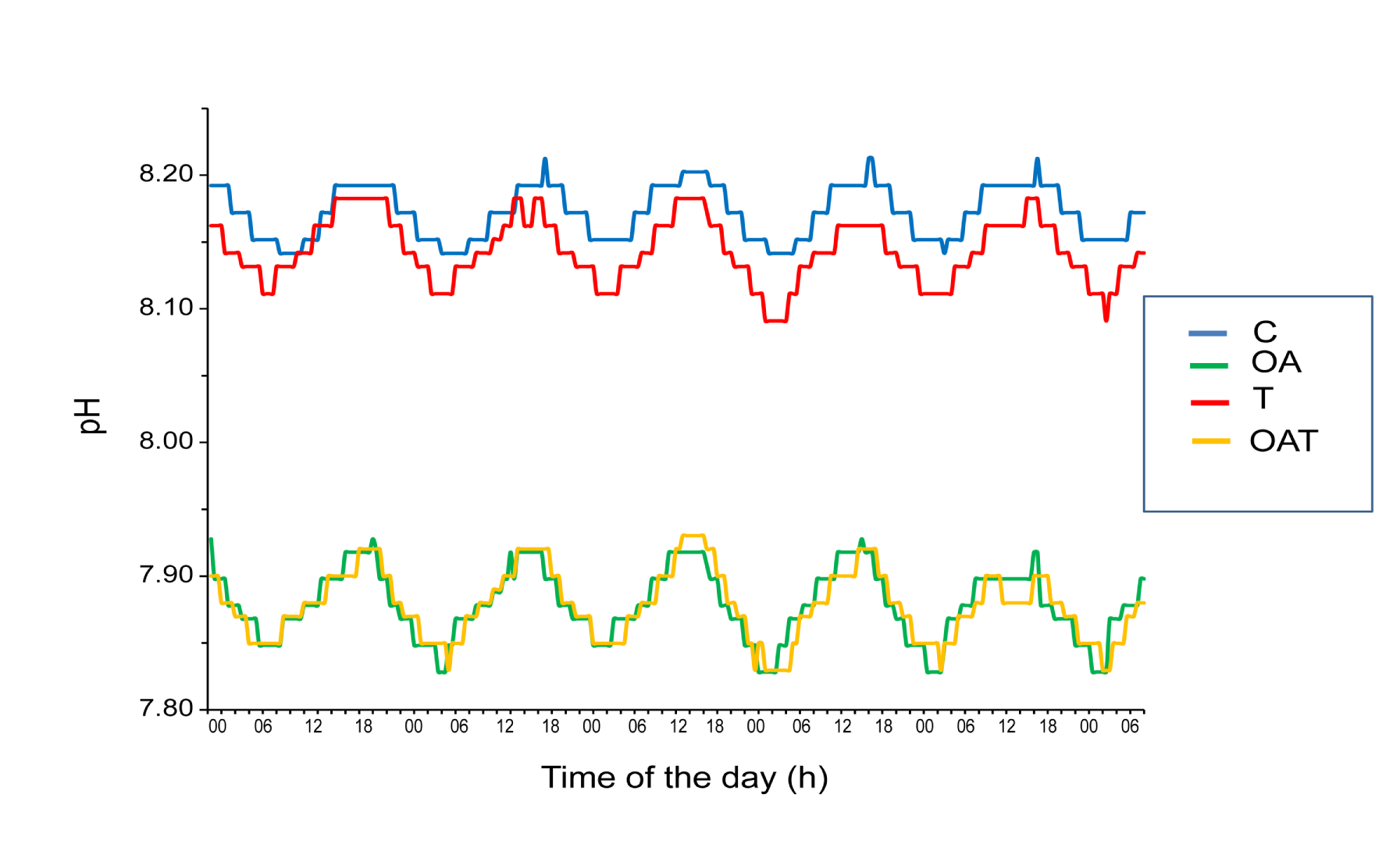

Supplement: S6 Fig — This analysis was only done for 1 mesocosm per treatment combination, serving as an example. For these 4 mesocosms in parallel, pH was recorded at 30-minute intervals with an automated pH logger (control system ACQ110 Aquatronica, Italy). (TIF) [file pbio.2003446.s006.tif]

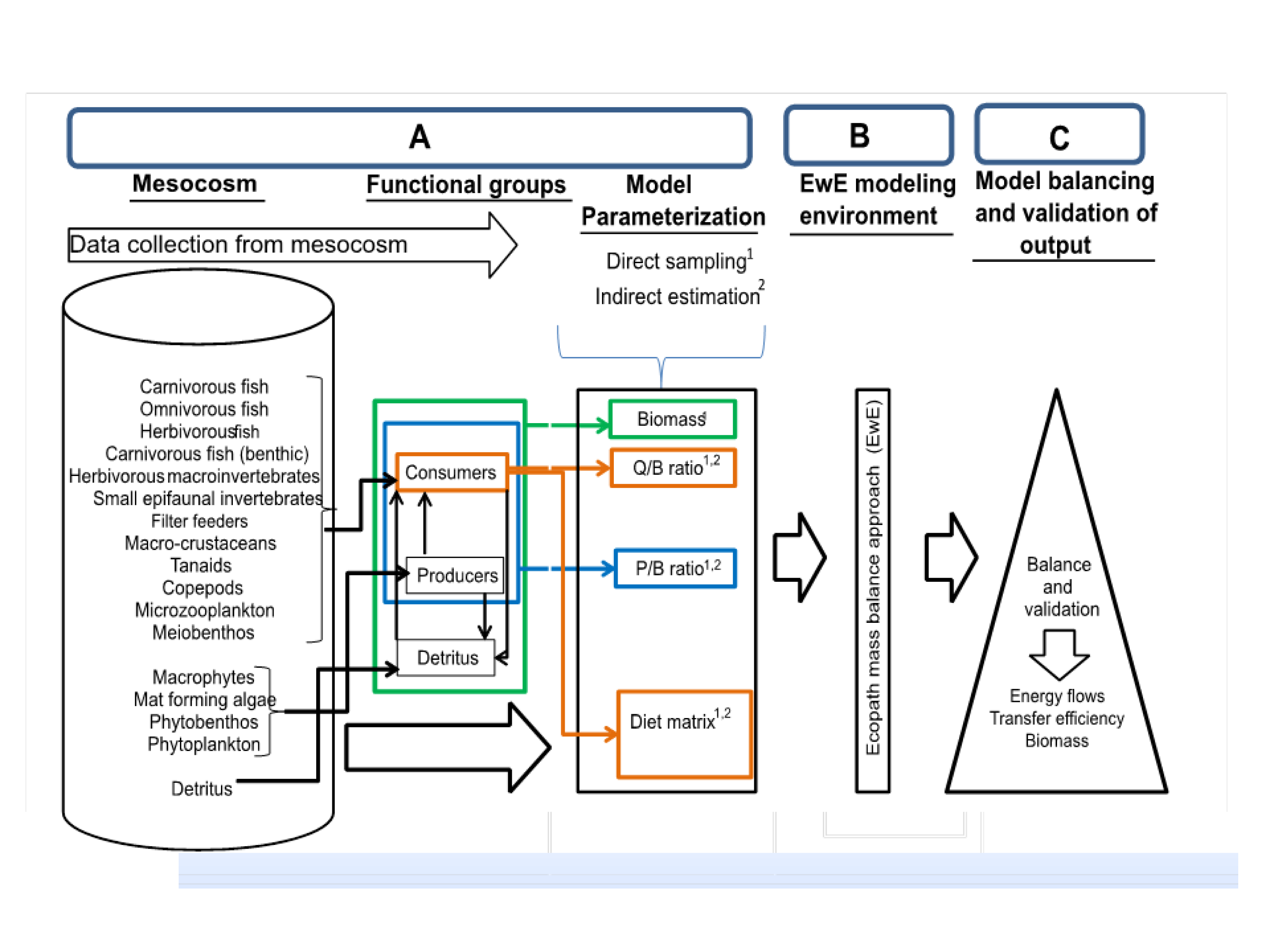

Supplement: S7 Fig — A) data collection from the mesocosms and parameter estimation, B) mass-balance modelling in Ecopath, and C) model balancing and validation. (TIF) [file pbio.2003446.s007.tif]

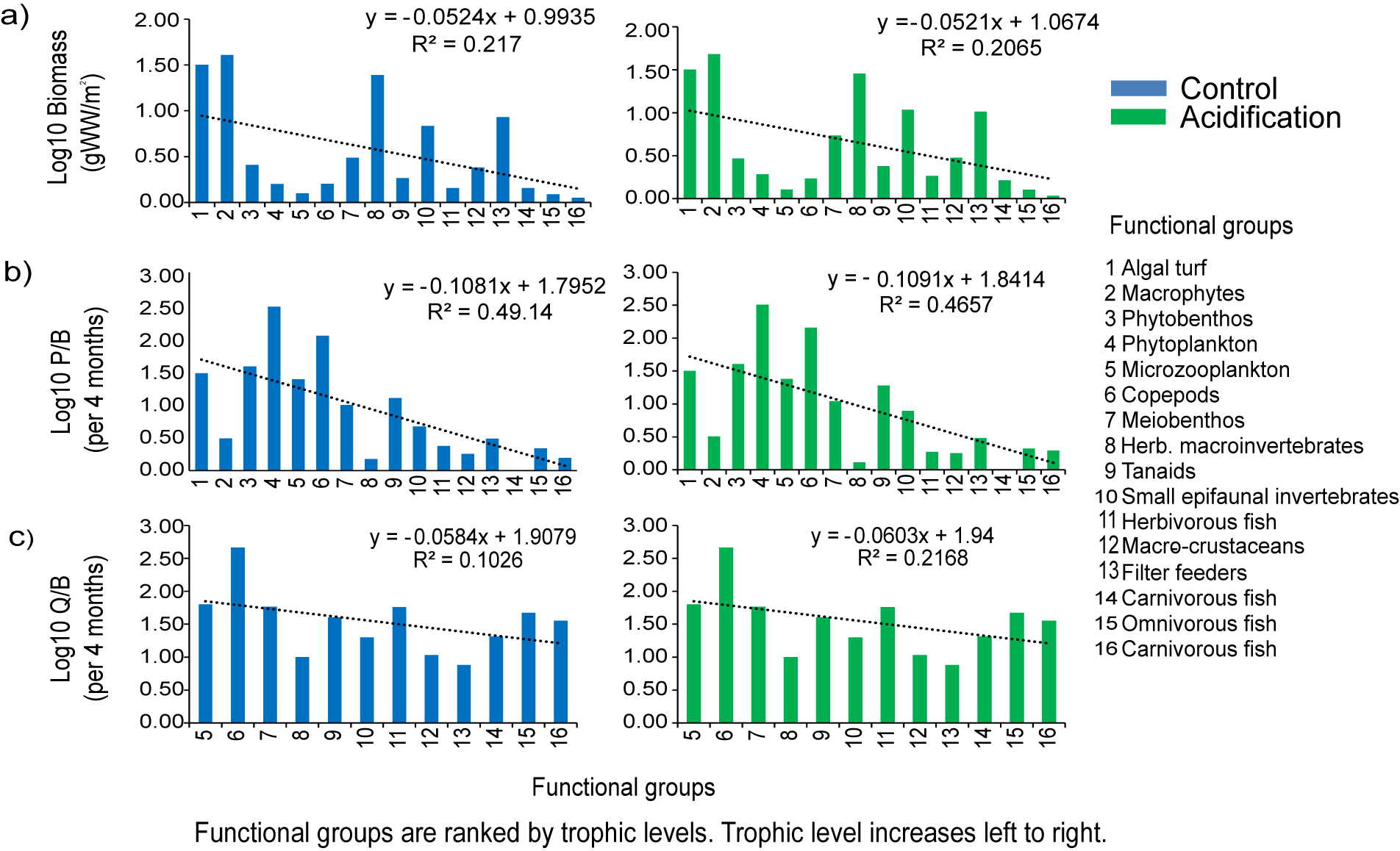

Supplement: S8 Fig — A constant of 1 was added to all response variables to avoid some negative values (Log10 [x + 1]) prior to PREBAL plotting. For specific functional group name, refer to the legend. Herb. = herbivorous. PREBAL is shown only for base models that are built on the average of all the input parameters (B, P/B, Q/B) across mesocosms within each climate treatment. B, biomass; P/B, production per unit of biomass; Q/B, consumption per unit of biomass. (TIF) [file pbio.2003446.s008.tif]

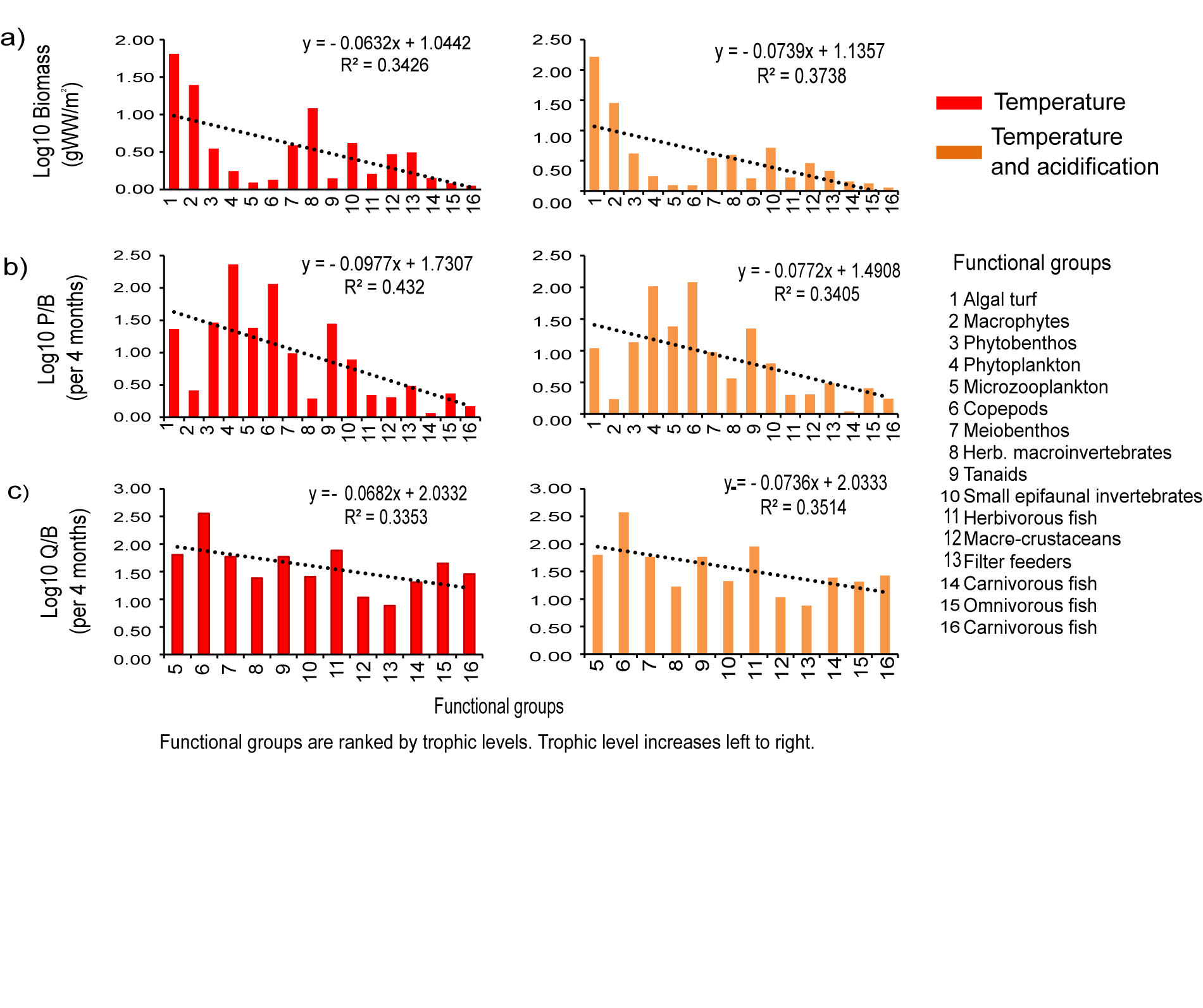

Supplement: S9 Fig — A constant of 1 was added to all response variables to avoid some negative values (Log10 [x + 1]) prior to PREBAL plotting. For specific functional group name, refer to the legend. Herb. = herbivorous. PREBAL is shown only for base models that are built on the average of all the input parameters (B, P/B, Q/B) across mesocosms within each climate treatment. B, biomass; P/B, production per unit of biomass; Q/B, consumption per unit of biomass. (TIF) [file pbio.2003446.s009.tif]
