# Supplementary material for: Climate change could drive marine food web collapse through altered trophic flows and cyanobacterial proliferation
Source: PLoS Biol. 2018 Jan 9;16(1):e2003446. doi: 10.1371/journal.pbio.2003446 (PMC5760012; doi:10.1371/journal.pbio.2003446)
Supplement: S1 Text — (DOCX) [file pbio.2003446.s026.docx]

Climate change could drive marine food web collapse through altered trophic flows and cyanobacterial proliferation

*Supplementary Methods*

**A. Data collection and parameter estimation**

Each of the twelve mesocosms had a surface area of 6.02 m^2^ with a water depth of 0.74 m. For the model parameterization, the vertical wall of our mesocosm was considered as an extended horizontal habitat since the mesocosm walls acted as rich habitat for benthic producers and served as a feeding ground for many of the mesocosms’ prey and predator communities. The total surface area considered per mesocosm, therefore, covered a true benthic habitat surface of 2.14 m^2^ (rocky reef = 0.60 m^2^; seagrass = 0.60 m^2^; sand = 0.92 m^2^) and a wall habitat of 3.89 m^2^.

Twelve food web models were built to represent the response of our mesocosms under different climate scenarios. Each climate treatment comprised three independent mesocosm food web models (see Table in S8,S9, S10 and S11 Tables for individual model descriptions). We used mesocosm-specific biomass data of each functional group for individual model inputs, whereas other input parameters (such as P/B, Q/B and diet data) were averaged across treatments. The food web models simulated a southern hemisphere summer period equivalent to 4 months (mid-March to mid-July 2015; maintained at treatment level. See S5 Fig for details). All parameters were standardized per unit surface area using wet weight (WW) for biomass (g WW.m^-2^), while energy flows are expressed per month. There are indeed various ways of expressing units of energy, one of which is carbon. However, in studies of marine ecosystems, energy flows are most often represented as wet weights. Furthermore, in our study, a substantial part of our data to quantify energy transfer rates was directly measured as wet weight. Therefore it is logical to use wet weight as the measure of unit for this experiment because it avoids uncertainty associated with converting the data to other measures.

The output (energy flow and transfer efficiency) of these food web models are presented in Fig 1. A schematic diagram shows the different phases of model building and execution ( S7 Fig).

The food web models consisted of 17 functional groups (ranging from primary producers to herbivores to carnivores across three trophic levels). Functional groups were categorized and grouped according to their similarities in ecological traits, such as feeding habits, size, habitat use, predator and life cycle [1].

We considered FishBase [2] and stomach content analysis (bio-volume) of the fishes in this study to allocate them to the different feeding guilds within the model. Two herbivorous fish species *Girella zebra* and *Acanthaluteres vittige* [2] partly consumed animal material which is not unusual for the juvenile life phase. However, they predominantly preyed on plant matter (~ 94%) in this experiment and therefore we considered them as herbivores. Each model included four functional groups of fish, two groups of invertebrates, one group of filter feeders, two groups of crustaceans, two groups of zooplankton, one group of benthic organisms, four groups of primary producers, and one detrital group.

We mostly used data from the mesocosms for estimating food web model parameters and creating the diet matrix for the different functional groups (Table in S12, S13, S14 and S15 Tables). In case of missing data for model parametrization, parameters were derived from empirical equations and the scientific literature (S16 Table).

**Fish identification and grouping:** At the beginning of the mesocosm experiment, fishes were placed onto a small tray with a ruler and then photographed with a camera (Canon EOS 60D). Every individual fish was measured for total length from the photographs using ImageJ software [3].

At the end of the experiment, individual fish length (total and standard length, cm) and wet weight were measured after carefully removing excess water by blotting with a paper towel. The weight of individual species for each mesocosm was summed, and biomass estimates were calculated as weight/area (g WW.m^-2^) for each mesocosm food web. Production/biomass (P/B) ratios are difficult to estimate directly. Therefore the P/B was considered to be equivalent to the instantaneous rate of total mortality (Z) [4], calculated as the negative natural logarithm of survival rate from the following equation,

$Z=-\ln(S)$ (4)

where *S* (total survival rate) = the number (*N*) of animals alive at the end of the experiment at time *t+1* divided by the number alive at the start of the experiment at time *t* and expressed as,

$S=(N_{t+1}/N_{t})$ (5)

Since our mesocosms are an unexploited (no fishing mortality) system, and there was no natural mortality for benthic carnivorous fish groups, initial P/B ratio was estimated as 0.001 (and not 0) for compatibility with Ecopath.

The food consumption per unit of biomass (Q/B) was estimated by dividing the average food consumption over the entire experimental period by average biomass gained by species/functional groups. Food consumption by fish species was calculated using feeding trials and based on 12 hours of feeding per day. The average biomass gained was calculated by deducting the average initial weight from the average final weight per species per mesocosm. The initial weight for each species was calculated using the length at the start of the experiment, based on an exponential growth model because it provided a better fit to the data compared to linear and non-linear growth models [5]. Therefore, the initial fish length was converted to wet weight using an exponential linear regression model derived from final length-weight data,

$y=\alpha e^{\beta x}$ (6)

where $y$ = weight at length “*x”*, $\alpha$ = the constant with a value of *x+1,* $\beta$ = rate of growth (when > 0) or decay (when < 0) and, *x* = length.

**Herbivorous macroinvertebrates:** Biomass of large herbivorous macroinvertebrates (e.g. *Bulla quoyii, Phasianella australis*, and *Thalotia conica*) was measured directly as wet weight (g WW.m^-2^) for each mesocosm at the end of the experiment. These macroinvertebrate species were considered for this model group due to their relatively large size (>1g). P/B ratios were calculated following Equation 4. The Q/B ratio was calculated by dividing the total food consumption over the entire experimental period by total species biomass. We assumed that the consumption rate was similar throughout time. Feeding trials were conducted to determine the food consumption rate of herbivorous macroinvertebrates. Ten quadrats were randomly placed per mesocosm (20 × 20 cm) and allowed to grow turf for a week without herbivorous snails. Then snails were allowed to feed on five quadrats for 24 hours. Quadrats with and without grazing were then collected and scraped off algal turf. Freshly collected turf hold a lot of water and create large variability in biomass measurements. Therefore, turf samples were first dried in an oven for 48 h at 65 °C and subsequently converted to wet weight using a standardised factor of 10 [6]. We converted dry weight back to wet weight as the latter was the parameter used for all other groups too. Feeding rate was calculated from the average turf weight of both grazed and non-grazed quadrats and used to calculate Q/B ratio for herbivorous macroinvertebrates in each mesocosm. The Q/B ratio calculated from the feeding trials for “acidification and temperature” treatments was not reliable due to the very low standing biomass of herbivorous macroinvertebrates (due to high treatment-related mortality), so we used the average of acidification and temperature treatment as a model input for “acidification and temperature”.

**Small epifaunal invertebrates:** Small epifaunal invertebrates comprise several groups of species such as small-sized herbivorous gastropods (average range 0.16-0.92 g; e.g., *Turbo undulates*, *Clanculus* sp*.*) occupying the open sandy habitat, and other small invertebrate herbivores (chitons, limpets, amphipods, juvenile abalone, juvenile sea urchins), detritivores (polychaetes, brittle stars), and predators (small sea stars and crabs) from the rocky reefs. Small-sized herbivores were counted, and their biomass was measured directly as wet weight to the nearest 0.1 g at the end of the experiment. Where individual animals were difficult to weigh due to their small size, individuals were pooled and weighed together as a group. The biomass of small epifaunal invertebrates was measured as wet weight (g WW.m^-2^).

It was difficult to directly estimate P/B and Q/B ratios for the small epifaunal invertebrates in our mesocosms. We therefore used data from a similar ecosystem [7] to assign a P/B value of 3.67 and Q/B value of 18 for small epifaunal invertebrates group in control conditions. The estimates come from the Te Tapuwae o Rongokako (TTMR) ecosystem model [8], which has a similar ecological and biological setting to our system, comprising a temperate coastal intertidal and subtidal ecosystem with both rocky reef and soft sediments. The predation pressure on small epifaunal invertebrates varied among the treatments. Since predation can cause an increase in population turnover rate [9] we adjusted P/B ratios for each treatment according to the predation pressure on small epifaunal invertebrates by consumers (fish) relative to the control tank. Accordingly, P/B ratio of the control treatment tank was multiplied by a factor of 1.94, 1.85, and 1.44 to calculate P/B ratio for acidification, temperature and acidification and temperature treatments, respectively. Since approximately 89% of small epifaunal invertebrate biomass comprised herbivorous species, we opted to use the same relative factors derived from macroinvertebrate consumption data to adjust Q/B ratio for small epifaunal invertebrates.

**Filter feeders:** Filter feeders (e.g. sponges, ascidians, tunicates, bivalves) were collected from rocky reefs (see above) and biomass was estimated as wet weight (g WW.m^-2^) for each mesocosm food web. We estimated the P/B and Q/B ratio using data from closely-related temperate marine systems [10-12]. We applied the same value across treatments because filter feeder biomass collapsed under the temperature and the combined temperature and acidification treatments.

**Shrimps:** Biomass of shrimps was estimated directly as final wet weight (g WW.m^-2^) for each mesocosm. The production over biomass (P/B) ratio and consumption over biomass (Q/B) ratio were obtained from empirical relationships following [13] and published sources for non-fish groups [8,14].

**Tanaids, copepods, and meiobenthos:** We collected tanaids, copepods, and meiobenthos samples twice from each mesocosm during the experimental period, using three types of benthic samplers (small cages), specifically designed to estimate 1) biomass (entirely open and accessible to predators for measurement of standing biomass), 2) production (covered with ~ 5 mm mesh size to exclude herbivores for measurement of production), and 3) as procedural controls (covered with mesh, but open at the sides, allowing predators to enter). Two replicate samplers of each of the three types were placed in each mesocosm and replicated over two time periods. The six samplers within each mesocosm at any one time were placed randomly keeping an equal distance from each other to reduce the likelihood of confounding factors. After extraction with Ludox TM colloidal solution, all tanaids, copepods, and meiobenthos from each sampler were counted under a stereomicroscope. Large tanaid shrimps (~ 2-5 mm in length) were weighed on a microscale (± 0.1 mg). For smaller-sized copepods (~ 0.2-1 mm long) and meiobenthos (~ 0.6-5.3 mm), a subsample (7.5% for each sample by randomly selecting 30 out of the 400 cells on a counting tray) was photographed to determine average individual mass (biovolume) using ImageJ. In total, 368 individuals from 3 groups were measured using ImageJ (copepods n=159, polychaetes and oligochaetes n=65, and nematodes n=138) to determine their biovolume. Only for copepods, the treatment-specific average individual mass was used to calculate total biomass since biovolume of copepods differed between climate treatments (ANOVA: F_(1,155)_=4.13, p=0.0438). Data were pooled for the two periods to get a representative biomass value for each of these functional groups.

The turnover rate (P/B) for tanaids and meiobenthos was calculated by dividing production over the experimental period by the standing biomass. We did not include data from one mesocosm (of the control treatment) to calculate biomass and production of meiobenthos due to a malfunction of the sampler. We used the same consumption rate per unit of biomass (Q/B) for tanaids and meiobenthos across treatments, based on [10]. The sampling method used to estimate copepod production did not work well due to the large mesh size used relative to copepod body sizes, and probably also due to the presence of carnivorous copepods inside the cage. Copepods are considered as one of the major prey items for shallow benthic carnivores as well as shallow water species [15]. We therefore used estimates of P/B and Q/B ratio for copepods from a closely related shallow, rocky reef dominated temperate marine ecosystem [8,16-18]. We assigned an average P/B value of 108.70 and Q/B value of 336.98 to parameterize the control model. We then adjusted the P/B ratio for other models based on the relative predation pressure on copepods by fishes compared to control treatments. Since production and consumption rate of functional groups is positively correlated, based on data provided in [18], Q/B ratio was further adjusted based on the corresponding P/B ratio through simple linear regression (Y = 3.5739X – 51.536; *R*^2^=0.98). We used the same P/B and Q/B ratio across all treatments.

Microzooplankton biomass was measured by filtering 400 L of water from each mesocosm through a plankton sampler at the end of the experiment. Initially, all samples were checked under a stereomicroscope to visually confirm the presence of a significant proportion of microzooplankton in the samples. Samples were rinsed through a 38 µm mesh sieve and then poured into a 100 ml measuring cylinder and allowed to settle for 24 hours, after which settling volume (ml) was recorded. Settling volumes were converted into displacement volumes using a factor of 0.35 for samples without gelatinous zooplankton [19]. Displacement volumes were converted to biomass (mg wet-weight) using a factor of 800 [20]. The P/B and Q/B ratio for microzooplankton was based on [8,11,12,17,21].

**Primary producer groups (phytoplankton, phytobenthos, mat-forming algae, macrophytes):** Four litres of water was filtered from each mesocosm with Whatman GF/C glass fiber filters of 4.7 cm diameter to estimate phytoplankton biomass. Phytobenthos samples were collected using the benthic samplers described earlier. The biomass (measured via Chlorophyll *a*) of both phytoplankton and phytobenthos was measured following [22]. The phytoplankton biomass value was converted into square meter units by multiplying by the euphotic depth (0.74 m). Both the phytoplankton and phytobenthos values were then converted to wet weight using two successive conversion factors [23]. The first was to change Chlorophyll *a* to carbon using a

40:1 – carbon to Chlorophyll *a* ratio

The second conversion factor was used to convert carbon to wet weight using

Wet weight = Carbon × 10

Macrophytes and mat-forming algal biomass were sampled from all habitats (rock, seagrass, and open sand) at the end of the experiment. All samples were dried in an oven at 65 °C for 24-48 hrs. Then, a conversion factor of 10 was applied to convert dry weight (g C.m^-2^) to wet weight (g WW.m^-2^) [6].

P/B ratios for primary producer groups were estimated by measuring the community photosynthesis of each mesocosm. First, we used published P/B ratios for phytoplankton [8,18,24], phytobenthos [8,24], mat-forming algae [25,26] and macrophytes [8,27] from closely related shallow temperate marine ecosystems to calculate a standard average P/B ratio for primary producer groups. We then used these ratios to estimate a relative production rate for each functional group and their corresponding P/B ratios from *in situ* community photosynthesis using the following equation (separately for each mesocosm),

$${\frac{P}{Bi}=\left\{ \frac{\left( \frac{aP}{Bi}\times B_{i} \right)\times100}{\sum\frac{aP}{Bi}\times B_{i}}\times\frac{\mathrm{CM}}{100} \right\}}/{Bi}$$

where $\frac{P}{Bi}$ is turnover rate calculated for group *i* as model input*,* $\frac{aP}{Bi}$ is the standard average turnover rate assigned for group *i,* $B_{i}$ is the biomass of group *i* sampled from each mesocosm*,* $\sum\frac{aP}{Bi}\times B_{i}$ is the total theoretical production for all functional groups, CM is the community photosynthesis measured *in situ* from the mesocosm, $\left\{ \frac{\left( \frac{aP}{Bi}\times B_{i} \right)\times100}{\sum\frac{aP}{Bi}\times B_{i}}\times\frac{\mathrm{CM}}{100} \right\}$ is the calculated production of functional group *i* to total community production measured in each mesocosm.

The average P/B value across treatments was used as the final model input. The assumption made here is that the variability in P/B ratio is only biomass driven. Thus the sum of all relative production (for the four producer groups) equals total community production.

Community photosynthesis (primary production) was measured as gross oxygen production (mg O_2_.m^-3^.min^-1^) based on production rates measured during daytime (net production) and consumption rate during night time (respiration):

Gross production = Net production + Respiration.

Each mesocosm had 14 hours of daytime and 10 hours of night time. The community photosynthesis was measured once per mesocosm at the end of the experiment. Mesocosms were sealed off from the atmosphere with a transparent plastic cover and seawater O_2_ concentration was measured at 1-min intervals over a 30 min period (HQ40d Portable Meter, sensor LDO101, HachTM). Both net community production and respiration values were transformed using following equation,

(mg O_2_.m^-2^.day^-1^) × 0 .375×2×10 = mg WW.m^-2^.day^-1^

where 0.375 is used to converts mass of oxygen to the mass of carbon and is a ratio of moles of carbon to moles of oxygen (12 mg C / 32 mg O_2_ = 0.375); 2 is multiplied to convert carbon to dry weight and then multiplied by 10 again for dry weight to wet weight. Finally, the gross production was calculated as g WW.m^-2^ per 4 months for initial model input.

**Detritus:** First, the benthic detritus layer was carefully separated from phytobenthos and zoobenthos (if any) from the top of the experimental samplers with a micro-spatula. Then the sediment samples were filtered through pre-combusted and pre-weighed Whatman GF/C glass fiber filters. Both the layer and extract were then oven-dried at 65 °C for 24-48 hrs. Finally, the dry weight was converted to wet weight (g WW.m^-2^) using a factor of 5 [28]. Detritus was considered to be particulate organic matter (POM) only.

**Diet composition of functional groups:** A diet matrix was constructed based on feeding rates measured in the final month of the experiment. Prior to measurement, all fishes were starved for 20 hrs and then released into their respective mesocosm for 4 hrs of free feeding before they were caught again and frozen immediately using a liquid nitrogen Dewar (-196 ^0^C) and placed in a cold freezer (-20 ^0^C) afterwards. Fish stomach contents were then analyzed under a stereo microscope by counting individual taxa (such as tanaid shrimp, copepods, bivalve shell and annelids) and weighing the total fish stomach content. The weight of different prey (g) was then calculated by multiplying the average individual body mass of corresponding prey to the count of each sample. Finally, the relative weight of different prey groups was calculated based on their relative contribution to the total prey weight and assigned for individual fish species. Diet composition of some functional groups was supplemented by local studies and relevant literature [8,10-12,17,27,29].

**Seagrass:** Previous lab studies found that maintaining seagrasses in indoor mesocosms is extremely difficult. We therefore had no other option than to use artificial seagrass to provide species with an important habitat for sheltering. This decision was made because habitat heterogeneity can have important implications for predator–prey interactions or heterospecific interactions [30,31] that are not captured by the model which is based on a homogenous environment. The predator–prey interactions are crucial to incorporate, to deliver a more realistic outcome at the food web level that can be strongly mediated by factors like habitat type and presence. We observed that these artificial seagrasses were frequently used by fish, shrimp and snails to obtain food and for sheltering purposes [32,33] and as such created a similar habitat to live seagrass beds that some of the species associate with in nature. The use of artificial seagrasses in itself is not expected to have major direct effects on the magnitude of energy flow or growth or turf algae and cyanobacteria.

**B. Mass-balance modelling in Ecopath**

Ecopath is a mass-balanced trophic model, which is grounded in general ecological theory [34-36]. Tests of Ecopath have proved the model capable of capturing real ecosystem dynamics in a variety of different ecosystem, ranging from temperate to tropical systems [37]. We used Ecopath to model the food webs in our mesocosms using linear equations for 17 functional groups. The parameterization of an Ecopath model is based on satisfying two ‘master’ equations: one for production (equation 1) and the other for the energy balance (equation 4)

The production of each group was calculated as ([1](#_ENREF_1)):

Production = predation mortality + biomass accumulation + net migration + other mortality (1)

and, written as:

$P_{i}=B_{i}\times M2_{i}+E_{i}+BA_{i}+P_{i}(1-EE_{i})$ (2)

where *Pi* is the total production rate of group (*i)*, Bi is the biomass of a group (*i)*, *M2i* is the instantaneous predation rate for group (*i)*, *Ei* is the net migration rate (emigration - immigration), *BAi* is the biomass accumulation rate for (*i)*, *EEi* is the ecotrophic efficiency of (*i)* describing the proportion of the production utilized in the system, and (1 − *EEi*) represents mortality other than predation.

This formula incorporates all the production (or mortality) except gonadal products which is assumed to be ending up being eaten by other groups, hence here considered under other mortality. Therefore Equation 2 can be expressed as:

$B_{i}(P/B)_{i}\times EE_{i}-\sum_{j=1}^{n} B_{j}(Q/B)_{j}\times DC_{ji}-E_{i}-BA_{i}=0$ (3)

where *P/Bi* is the production/biomass ratio for (*i),* Q*/Bj* is the consumption/biomass ratio of the predator ( *j)* and *DCji* is the fraction of prey (*i)* in the average diet of predator (*j)*. All other variables are the same as those described for Equation 2.

The energy input and output of all living groups is balanced using equation (4):

Consumption = production + respiration + unassimilated food (4)

and, written as:

$Q_{i}=Pi+Ri+UAi$ (5)

where Qi is consumption by a group (*i)*, *Pi* is total production of group (*i)*, *Ri* is respiration of a group (*i)* and *UAi* is the unassimilated food of group (*i)*.

**C. Model balancing and validation**

Prior to model balancing, a set of pre-balancing (PREBAL) analyses was used to assess whether data abide by the general rules/principles of ecosystem ecology [38]: (i) biomass of functional groups should span 5–7 orders of magnitude when arranged against their trophic levels; (ii) the slope of biomass (on a logarithmic scale) by functional groups should decline by 5–10% across all the taxa when arranged against trophic levels; (iii) there is a general decline of vital rates (P/B; Q/B) across taxa/trophic levels with increasing trophic level [7,38]. Our tests showed that these general ecological rules were met by our model (S8 andS9 Figs).

We applied a manual mass-balanced procedure, using a ‘top-down’ approach (starting with the top predator groups and moving down the food web to balance inconsistencies) adjusting the input parameters of those groups ‘out of balance’ (EE > 1). The ecological models were considered balanced when the following thermodynamic and ecological rules were met [7,39].

1. Ecotrophic efficiency (EE) < 1. EE is a measure of the proportion of production that is utilized by the next trophic level through direct predation. The value for EE can never exceed 1.0 as it is not possible for more production to be passed on to the next trophic level than was originally produced.
2. Gross food conversion efficiency (GE) between 0.1 and 0.35.
3. Net efficiency > GE. Net efficiency is the value for food conversion after accounting for unassimilated food. GE can never exceed Net efficiency.
4. Respiration/assimilation biomass (RA/A) ratio < 1.0. The proportion of biomass lost through respiration cannot exceed the biomass of food assimilated.
5. Production/respiration (P/RA) ratio < 1.0. This ratio expresses the fate of assimilated food.

To achieve mass balance, we modified parameters with the highest levels of uncertainty, such as the diet matrix. Since biomass estimates were based on high-precision sampling, they were not modified. To obtain mass balance, we adjusted the diet matrix of non-fish groups such as filter feeders for the control and temperature models and small epifaunal cryptic invertebrates, filter feeders, tanaids and copepods for acidification and combined acidification and temperature models. We also had to manually adjust the diet data for omnivorous fishes in the temperature and combined acidification and temperature models. This is because diets of omnivorous fish are difficult to estimate by using gut content data alone [40]. We had to adjust P/B or Q/B ratio for tanaids to lower down the expected range for temperature and acidification and temperature models [1]. A model default value of 0.2 (dimensionless) was set for unassimilated consumption rate for all groups, except zooplankton where 0.3 was used [8]. Once balanced, EE values were < 1 for all functional groups confirming that the model fulfilled the first basic requirement of thermodynamic and ecological rules. The net efficiency was also lower than the gross food conversion efficiency. The gross food conversion efficiency (P/Q), production/respiration (P/RA) and the respiration over assimilation (RA/A) were within the expected ranges [1]. The resulting output parameters and the final diet matrix are shown for each model (S8, S9, S10, S11, S12, S13, S14 and S15 Tables).

**Pedigree index and quality of the model:** The robustness of our Ecopath models was assessed through a ‘Pedigree index.' The pedigree of a model addresses the problem of propagating uncertainty, providing an index of model confidence. Pedigree values were assigned to each parameter for each group and then an overall model ‘Pedigree index,' P, was calculated:

$$P=\sum_{i=1}^{n} \frac{I_{i,j}}{n}$$

where *I_i,p_* is the pedigree index value for group *i* and input parameter *j* for each of the *n* living groups in the ecosystem; *j* can represent either B, P/B, Q/B, Y or the diet composition [1].

The P scales between 0 and 1 (inclusive). Models with a pedigree value ≥ 0.6 are considered robust [41]. The pedigree index P is, however, a function of the number of groups in the system. We therefore also calculated an overall measure of fit, *t**

$$t^{*}=P\times\frac{\sqrt{n-2}}{\sqrt{1-P^{2}}}$$

where *n* is the number of living groups in the given model.

The pedigree index for each of our mesocosm models was 0.71 (a measure of fit *t*=3.819), indicating that our models are robust [1,42].

**References**

1. Christensen V, Walters C, Pauly D (2005) Ecopath with Ecosim: A User's guide. Vancouver, BC: Fisheries Centre, University of British Columbia. 154pp. p.

2. Froese R FishBase.

3. Schneider CA, Rasband WS, Eliceiri KW (2012) NIH Image to ImageJ: 25 years of image analysis. Nat Meth 9: 671-675.

4. Allen KR (1971) Relation Between Production and Biomass. Journal of the Fisheries Research Board of Canada 28: 1573-1581.

5. Scharf FS (2000) Patterns in Abundance, Growth, and Mortality of Juvenile Red Drum across Estuaries on the Texas Coast with Implications for Recruitment and Stock Enhancement. Transactions of the American Fisheries Society 129: 1207-1222.

6. Bundy A (2004) Mass balance models of the eastern Scotian Shelf before and after the cod collapse and other ecosystem changes: Department of Fisheries and Oceans.

7. Heymans JJ, Coll M, Link JS, Mackinson S, Steenbeek J, et al. Best practice in Ecopath with Ecosim food-web models for ecosystem-based management. Ecological Modelling <http://dx.doi.org/10.1016/j.ecolmodel.2015.12.007>.

8. Pinkerton MH, Lundquist CJ, Duffy CAJ, Freeman DJ (2008) Trophic modelling of a New Zealand rocky reef ecosystem using simultaneous adjustment of diet, biomass and energetic parameters. Journal of Experimental Marine Biology and Ecology 367: 189-203.

9. Chesney EJ (1985) Laboratory studies of the effect of predation on production and the production:biomass ratio of the opportunistic polychaete Capitella capitata (Type I). Marine Biology 87: 307-312.

10. Wilson R, Cohen B, Poore GC (1993) The role of suspension-feeding and deposit-feeding benthic macroinvertebrates in nutrient cycling in Port Phillip Bay.

11. Goldsworthy SD, Page B, Rogers PJ, Bulman C, Wiebkin A, et al. (2013) Trophodynamics of the eastern Great Australian Bight ecosystem: Ecological change associated with the growth of Australia's largest fishery. Ecological Modelling 255: 38-57.

12. Watson RA, Nowara GB, Tracey SR, Fulton EA, Bulman CM, et al. (2013) Ecosystem model of Tasmanian waters explores impacts of climate-change induced changes in primary productivity. Ecological Modelling 264: 115-129.

13. Brey T (2001) Population dynamics in benthic invertebrates. A virtual handbook. htpp. www awi-bremerhaven de/Benthic/Ecosystem/FoodWeb/Handbook/main html Alfred Wegener Institute for Polar and Marine Research, Germany.

14. Cheng J, Cheung WWL, Pitcher TJ (2009) Mass-balance ecosystem model of the East China Sea. Progress in Natural Science 19: 1271-1280.

15. Kramer MJ, Bellwood O, Bellwood DR (2013) The trophic importance of algal turfs for coral reef fishes: the crustacean link. Coral Reefs 32: 575-583.

16. Beattie G, Redden A, Royle R (1996) Microzooplankton grazing on phytoplankton in Port Phillip Bay: CSIRO Environmental Projects Office.

17. Lozano-Montes HM, Loneragan NR, Babcock RC, Jackson K (2011) Using trophic flows and ecosystem structure to model the effects of fishing in the Jurien Bay Marine Park, temperate Western Australia. Marine and Freshwater Research 62: 421-431.

18. Kolding J, Bundy A, van Zwieten PAM, Plank MJ (2016) Fisheries, the inverted food pyramid. ICES Journal of Marine Science 73: 1697-1713.

19. Wiebe PH (1988) Functional regression equations for zooplankton displacement volume, wet weight, dry weight, and carbon: a correction. Fish Bull 86: 833-835.

20. Cushing D, Humphrey G, Banse K, Laevastu T (1958) Report of the committee on terms and equivalents. Rapp P-V Reun Cons Int Explor Mer 144: 15-16.

21. Fulton E, SMITH AD (2004) Lessons learnt from a comparison of three ecosystem models for Port Phillip Bay, Australia. African Journal of Marine Science 26: 219-243.

22. Jeffrey SW, Humphrey GF (1975) New spectrophotometric equations for determining chlorophylls a, b, c1 and c2 in higher plants, algae and natural phytoplankton. Biochemie und Physiologie der Pflanzen 167: 191-194.

23. Jones JG (1979) Guide to methods for estimating microbial numbers and biomass in fresh water. Guide to methods for estimating microbial numbers and biomass in fresh water: Freshwater Biological Association.

24. Murray A, Parslow J (1997) Port Phillip Bay integrated model: final report: Port Phillip Bay Environmental Study, CSIRO Environmental Projects Office.

25. Bozec Y-M, Gascuel D, Kulbicki M (2004) Trophic model of lagoonal communities in a large open atoll (Uvea, Loyalty islands, New Caledonia)*. Aquat Living Resour 17: 151-162.

26. Wabnitz CCC, Balazs G, Beavers S, Bjorndal KA, Bolten AB, et al. (2010) Ecosystem structure and processes at Kaloko Honokōhau, focusing on the role of herbivores, including the green sea turtle Chelonia mydas, in reef resilience. Marine Ecology Progress Series 420: 27-44.

27. Dommasnes A, Christensen V, Ellertsen B, Kvamme C, Melle W, et al. An Ecopath model for the Norwegian Sea and Barents Sea.

28. Strickland J (1966) Measuring the production of marine phytoplankton.

29. Cornwall CE, Eddy TD (2015) Effects of near-future ocean acidification, fishing, and marine protection on a temperate coastal ecosystem. Conservation Biology 29: 207-215.

30. Oksanen T (1990) Exploitation ecosystems in heterogeneous habitat complexes. Evolutionary Ecology 4: 220-234.

31. Ljungberg P, Hasper TB, Nilsson PA, Persson A (2013) Effects of small-scale habitat fragmentation on predator–prey interactions in a temperate sea grass system. Marine Biology 160: 667-675.

32. Adams AJ, Locascio JV, Robbins BD (2004) Microhabitat use by a post-settlement stage estuarine fish: evidence from relative abundance and predation among habitats. Journal of Experimental Marine Biology and Ecology 299: 17-33.

33. Bell JD, Steffe AS, Westoby M (1985) Artificial seagrass: How useful is it for field experiments on fish and macroinvertebrates? Journal of Experimental Marine Biology and Ecology 90: 171-177.

34. Polovina JJ (1984) Model of a coral reef ecosystem. Coral Reefs 3: 1-11.

35. Walters C, Christensen V, Pauly D (1997) Structuring dynamic models of exploited ecosystems from trophic mass-balance assessments. Reviews in Fish Biology and Fisheries 7: 139-172.

36. Christensen V, Pauly D (1992) ECOPATH II — a software for balancing steady-state ecosystem models and calculating network characteristics. Ecological Modelling 61: 169-185.

37. Walters CJ, Christensen V, Martell SJ, Kitchell JF (2005) Possible ecosystem impacts of applying MSY policies from single-species assessment. ICES Journal of Marine Science 62: 558-568.

38. Link JS (2010) Adding rigor to ecological network models by evaluating a set of pre-balance diagnostics: A plea for PREBAL. Ecological Modelling 221: 1580-1591.

39. Darwall WRT, Allison EH, Turner GF, Irvine K (2010) Lake of flies, or lake of fish? A trophic model of Lake Malawi. Ecological Modelling 221: 713-727.

40. Carassou L, Kulbicki M, Nicola TJR, Polunin NVC (2008) Assessment of fish trophic status and relationships by stable isotope data in the coral reef lagoon of New Caledonia, southwest Pacific. Aquat Living Resour 21: 1-12.

41. Morissette L (2007) Complexity, cost and quality of ecosystem models and their impact on resilience: a comparative analysis, with emphasis on marine mammals and the Gulf of St. Lawrence: University of British Columbia.

42. Christensen V, Walters CJ (2004) Ecopath with Ecosim: methods, capabilities and limitations. Ecological Modelling 172: 109-139.
